# A synopsis of the scorpion fauna of French Guiana, with description of four new species

**DOI:** 10.3897/zookeys.764.25108

**Published:** 2018-06-05

**Authors:** Eric Ythier

**Affiliations:** 1 SynTech Research, 613 route du Bois de Loyse, 71570 La Chapelle de Guinchay, France

**Keywords:** *Ananteris*, *Auyantepuia*, French Guiana, geographical distribution, *Hadrurochactas*, key, new scorpion species, synopsis

## Abstract

A synopsis is provided for all scorpion species collected in French Guiana, including thorough diagnoses and additional distributional records for each documented species. Four new species are also described in this paper (one *Ananteris* from northeastern Guiana, two *Auyantepuia* from central and northeastern Guiana and one *Hadrurochactas* from western Guiana), raising the total number of species described from French Guiana to 30. Most of the species are illustrated, geographical distribution maps are presented, and a key to the species is proposed.

## Introduction

Since the comprehensive work of Lourenço on the scorpion fauna of French Guiana published in 1983, several genera, species, and subspecies were described, few species were synonymized or transferred to other genera and additional collection material increased our knowledge of the geographical distribution of several species. Lourenço (1983) documented a total of 18 species belonging to eight genera and three families, while the present paper records a total of 30 species belonging to 12 genera and three families. All new species described since 1983 were by Lourenço (eight species; 1997b, 2001b, 2003b, 2008, 2012c, 2012d, 2016a, 2016b), Lourenço and Monod (one species; 1999), Lourenço and Ythier (one species; 2011) and Ythier (five species; 2015 and present paper).

As discussed in several publications (Lourenço 1986, 1991, 2001a, 2016a, 2016b) the scorpion fauna of the Guianan region (Guayana floristic province as described in Mori 1991) presents a great complexity of endemism. The Guianan rainforests are one of the largest continuous tracts of relatively pristine lowland tropical rainforest in the World. This ecoregion is characterized by high species richness and local and regional endemism, as well as relatively intact ecological processes. Species assemblages are shared with the Amazon and Orinoco basins, and with the Guianan highlands and Tepuy formations, and the ecoregion is thus a convergence zone for species diversity. The degree of endemism for the scorpion species present in the region is high, with 21 of the 30 species presented in this paper possibly endemic to French Guiana. Despite its reasonably small size of 83,000 km², French Guiana is a biodiversity hotspot presenting remarkable scorpion diversity and the present paper probably represents only a small part of the scorpion fauna actually present, since most of the region has not been studied yet and several specific areas with potentially high degrees of endemism (e.g., inselbergs) have only been recently started to be studied. For several sections of the lowland rainforest such as the canopy, knowledge of the scorpion fauna is also still almost nonexistent.

French Guiana has a wide range of habitats (Fig. 33). Most of its land area (90%) is covered by lowland tropical rainforest (Fig. 34) interspersed with forests of higher altitude (Fig. 35), 95% of these rainforests consisting or primary forest. Along the coast there are also patches of dry forest including white sand forests (Fig. 36) and savannas (Fig. 37), as well as many types of wetlands including mangroves and swamps. While several species are commonly found in many different habitats across French Guiana (e.g., *T. obscurus*, *T. silvestris*, *B. gervaisii*, *B. granulatus*) including zones impacted by human activities, some others remain endemic elements from specific ecosystems like e.g., coastal dry forests or savannas (e.g., *A. elisabethae*, *A. kalina* sp. n., *J. pintoi kourouensis*, *O. heurtaultae*, *T. mana*) or French Guiana massifs represented by inselbergs (e.g., *A. polleti*, *A. sabineae*, *S. mitaraka*).

## Materials and methods

Material presented herein is deposited in the following collections:

**MNHN** Muséum national d’Histoire naturelle, Paris, France,

**MHNG** Muséum d’histoire naturelle, Geneva, Switzerland,

**EYPC** Eric Ythier Private Collection, Romanèche-Thorins, France,

**RNA** Reserve Naturelle de l’Amana, Awala-Yalimapo, French Guiana, France.

With the exception of the specimens deposited in the MHNG, most of the presented material was examined by the author. For each species documented in this work, geographical distribution is presented based on the studied material (see material section for each species and corresponding distribution maps in Figs 31, 32). Distribution data for specimens not deposited in one of the above-mentioned collections was not taken into account.

Thorough diagnoses are presented for the species described previously to this note, extracted from original descriptions and in some cases translated from French, German, Spanish, and Portuguese. For detailed descriptions or additional information, readers can refer to the publications indicated in the references. Photographs of alive or fixed specimens are presented, when available. Measurements and illustrations were made using a Motic DM143 digital stereo-microscope together with a Nikon D810 camera and a Wacom Intuos drawing tablet. Measurements follow Stahnke (1970) and are given in mm. Trichobothrial notations are those developed by Vachon (1974) and the morphological terminology mostly follows Hjelle (1990).

### List of scorpion species from French Guiana

Family Buthidae C. L. Koch, 1837

Genus *Ananteris* Thorell, 1891

*Ananteris coineaui* Lourenço, 1982 (*)

*Ananteris elisabethae* Lourenço, 2003 (*)

*Ananteris guyanensis* Lourenço & Monod, 1999 (*)

*Ananteris intermedia* Lourenço, 2012 (*)

*Ananteris kalina* sp. n. (*)

*Ananteris polleti* Lourenço, 2016 (*)

*Ananteris sabineae* Lourenço, 2001 (*)

Genus *Isometrus* Hemprich & Ehrenberg, 1828

*Isometrus maculatus* (DeGeer, 1778)

Genus *Jaguajir* Esposito, Yamaguti, Souza, Pinto da Rocha & Prendini, 2017

*Jaguajir pintoi kourouensis* (Lourenço, 2008)

Genus *Microananteris* Lourenço, 2003

*Microananteris minor* Lourenço, 2003 (*)

Genus *Tityus* C. L. Koch, 1836

Tityus (Tityus) gasci Lourenço, 1981

Tityus (Archaeotityus) mana Lourenço, 2012 (*)

Tityus (Atreus) obscurus (Gervais, 1843)

Tityus (Archaeotityus) silvestris Pocock, 1897

Family Chactidae Pocock, 1893

Genus *Auyantepuia* Gonzalez-Sponga, 1978

*Auyantepuia aluku* sp. n. (*)

*Auyantepuia aurum* sp. n. (*)

*Auyantepuia fravalae* Lourenço, 1983 (*)

*Auyantepuia gaillardi* Lourenço, 1983 (*)

*Auyantepuia kelleri* Lourenço, 1997 (*)

*Auyantepuia laurae* Ythier, 2015 (*)

*Auyantepuia sissomi* Lourenço, 1983 (*)

Genus *Broteochactas* Pocock, 1893

*Broteochactas delicatus* (Karsch, 1879)

Genus *Brotheas* C. L. Koch, 1837

*Brotheas gervaisii* Pocock, 1893

*Brotheas granulatus* Simon, 1877

Genus *Guyanochactas* Lourenço, 1998

*Guyanochactas flavus* Lourenço & Ythier, 2011 (*)

*Guyanochactas gonzalezspongai* (Lourenço, 1983) (*)

Genus *Hadrurochactas* Pocock, 1893

*Hadrurochactas cristinae* sp. n. (*)

*Hadrurochactas schaumii* (Karsch, 1880)

Genus *Spinochactas* Lourenço, 2016

*Spinochactas mitaraka* Lourenço, 2016 (*)

Family Hormuridae Laurie, 1896

Genus *Opisthacanthus* Peters, 1861

*Opisthacanthus heurtaultae* Lourenço, 1980 (*)

Total 30 species. 21 (*) are possibly endemic elements to French Guiana. Other species also occur in Brazil (*J. pintoi*, *T. gasci*, *T. obscurus*, *T. silvestris*, *B. delicatus*, *B. gervaisii*, *B. granulatus*), Peru (*T. gasci*, *T. silvestris*), Ecuador (*T. gasci*), Suriname (*T. obscurus*, *T. silvestris*, *H. schaumii*), Guyana (*R. pintoi*, *H. schaumii*), Venezuela (*H. schaumii*) or have cosmopolitan distribution (*I. maculatus*).

## Taxonomic treatment

### Family BUTHIDAE C. L. Koch, 1837

#### Genus *Ananteris* Thorell, 1891

##### Ananteris coineaui

Taxon classificationAnimaliaScorpionesButhidae

Lourenço, 1982

Fig. 1

###### References.

Lourenço 1982, Lourenço and Cuellar 1999, Fet et al. 2000, Lourenço 2003a.

###### Material.

Downstream from Saut Pararé on Arataye river, Approuague tributary, one female (holotype), MNHNRS8504, J.P. Gasc coll., IV-V/1979. Right bank of Arataye river, downstream from Saut Pararé, in a palm tree (*Astrocaryum paramaca*) in forest, two females (paratypes), J.P. Gasc coll., I/1981. Saül, in a palm tree, one female, deposited in the MNHN, W. Lourenço leg.

###### Diagnosis.

Species of medium to large size compared to the average size of the other species within the genus (32.7 mm in total length for female holotype). General coloration dark yellow with brown to dark brown variegated pigmented zones. Carapace dark yellow with dark brown spots mainly on the anterior edge; lateral and posterior edges with less spots; eyes surrounded by black pigment. Mesosoma dark yellow with confluent brownish spots on all tergites; the VII with a triangular brownish spot; the lateral edges with square spots with the center lighter. Venter yellow ochre to brownish, with dark brown spots on edges of sternites V to VII, especially on the VII. Metasomal segments I to III reddish yellow, IV and V reddish; one triangular brownish spot on I to IV, dorsally. Vesicle reddish yellow, lighter than metasomal segment V; base of aculeus yellowish, tip reddish. Chelicerae yellow with a dark brown spot anteriorly, at the base of fingers; fingers reddish black. Pedipalps dark yellow; femur, patella, and chela strongly marked with dark brown spots; chela almost entirely dark brown; fingers reddish yellow. Legs reddish yellow, with numerous dark brown spots. Carapace with moderately marked granulation; anterior margin almost straight; all furrows moderate to weak. Tergites with moderately marked granulation, similar to that of carapace, better marked posteriorly; median carina well-marked on all tergites. Pectinal tooth count 16–16 to 17–17 in female. Sternites with spiracles linear. Metasomal segments with 10-10-8-8-5 crenulate carinae; intercarinal spaces strongly granular on all segments; segment V rounded. Vesicle with some granulation lateraly; subaculear tooth strongly marked. Pedipalp femur pentacarinate; patella and chela with weak to vestigial carinae; movable fingers with seven linear rows of granules.

**Figure 1.**
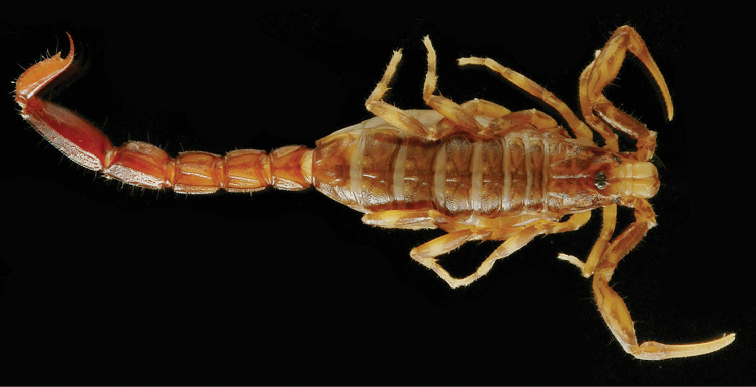
*Ananteris coineaui*, female holotype from Saut Pararé (photo MNHN / E.-A. Leguin).

##### Ananteris elisabethae

Taxon classificationAnimaliaScorpionesButhidae

Lourenço, 2003

###### References.

Lourenço 1983, Lourenço 1993, Lourenço 2003a, Lourenço 2016b.

###### Material.

Kourou, in the forest, one male (holotype), MNHNRS8086, mission M. Boulard & P. Pompanon coll., 37/VIII/1975.

###### Diagnosis.

Species of small size when compared with the average size of the other species of the genus (17.7 mm in total length for male holotype). Generally pale yellow without any spots or pigmented zones on the body and its appendages. Carapace yellowish; only the eyes surrounded by black pigment. Mesosoma yellowish with some pale reddish zones on the posterior edges of tergites. Venter pale yellow. All segments of metasoma yellowish; segment V slightly darker. Vesicle yellowish and aculeus yellowish. Chelicerae and teeth globally yellowish. Pedipalps yellowish overall, including the rows of granules on the dentate margins of the fingers. Legs yellowish. Carapace moderately to weakly granular; anterior margin weakly emarginated; all furrows moderate to weak. Tergites moderate to weakly granular; median carina moderate to weak in all tergites; tergite VII pentacarinate. Pectinal tooth count 16–16 in male. Sternites weakly granular with moderately elongate stigmata; VII with vestigial carinae. Metasomal segments with 10-8-8-8-5 crenulate carinae; intercarinal spaces weakly granular; segment V rounded. Telson elongated and weakly granular with three ventral carinae; the latero-ventral vestigial; aculeus short and moderately curved; subaculear tooth strong and spinoid. Pedipalp femur pentacarinate; patella and chela with a few vestigial carinae; internal aspect of patella with 7–8 spinoid granules; all aspects weakly granular, almost smooth; fixed and movable fingers with six almost linear rows of granules; two small accessory granules present at the base of each row. Leg tarsus with very numerous fine median setae ventrally; tibial spurs strongly developed on legs III and IV.

##### Ananteris guyanensis

Taxon classificationAnimaliaScorpionesButhidae

Lourenço & Monod, 1999

Fig. 2

###### References.

Lourenço and Monod 1999, Fet et al. 2000, Lourenço 2003a, Lourenço 2016b.

###### Material.

Saint Eugène, rainforest, in rotten log, one female (holotype), deposited in the MHNG, R. Boistel leg., 15/IV/1998. Mana, near Saut Sabbat, Gîte Angoulème, one male, deposited in the EYPC, EY0106, J. Chevalier & B. Tan coll., 04/XI/2017. Mana, near Saut Sabbat, Gîte Angoulème, one female, deposited in the RNA, J. Chevalier & B. Tan coll., 07/I/2018.

###### Diagnosis.

Species of medium size when compared with the average size of the other species of the genus, ranging from 25 to 30 mm in total length. General coloration yellowish brown, symmetrically marbled with dark reddish brown, producing an overall spotted appearance. Carapace yellowish brown and heavily spotted; eyes surrounded with black pigment. Mesosoma yellowish brown with confluent brown stripes and two longitudinal yellowish stripes. Venter yellowish with spots only on sternite VII. Metasomal segments I to V yellowish brown, with numerous brown spots; segments IV and V reddish, darker than the others. Vesicle reddish yellow without spots, but with some darker areas over the carinae. Chelicerae yellowish without variegated spots over their entire surface, and with only a dark thin zone at the base of the fingers; fingers reddish. Pedipalps dark brown with spots on the femur and patella; chelae yellowish; fingers brownish. Legs brownish with fuscous spots. Carapace feebly to moderately granular; anterior margin with a slight median concavity; all furrows moderate to feeble. Tergites moderately granular; median carina moderate to strong in all tergites. Pectinal tooth count 17–16 in female. Sternites almost smooth with moderate elongate stigmata; VII with four vestigial carinae. Metasomal segments with 10-10-10-8-5 crenulate carinae; intercarinal spaces moderately granular; segment V rounded. Telson moderately granular with one ventral carina and with a fairly short and moderately curved aculeus; subaculear tooth strong and spinoid. Pedipalp femur pentacarinate; patella and chelae with a few carinae but moderately crenulate; internal side of patella with only vestigial spinoid granules; all sides moderately to feebly granular; movable fingers with seven oblique rows of granules; only one accessory granule present at the base of each row. Leg tarsus with very numerous fine median setae ventrally; tibial spurs strongly developed on legs III and IV.

**Figure 2.**
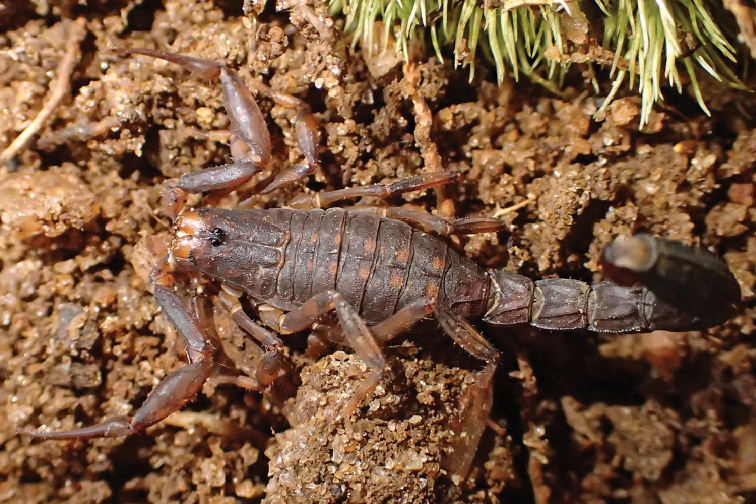
*Ananteris guyanensis*, female from Saut Sabbat (photo J. Chevalier).

##### Ananteris intermedia

Taxon classificationAnimaliaScorpionesButhidae

Lourenço, 2012

Fig. 3

###### References.

Lourenço 2012c.

###### Material.

St. Jean du Maroni, road to Saint Laurent, primary forest, winkler, one male (holotype), deposited in the MNHN, W. Lourenço coll., 12/VI/1987.

###### Diagnosis.

Very small species when compared with the average size of the other species of the genus (9.3 mm in total length for male holotype). General coloration yellow to pale yellow with carapace and tergites intensely marbled with dark brown spots, producing an overall spotted appearance. Carapace yellow, almost totally covered with brown spots; eyes surrounded by black pigment. Mesosoma yellowish brown with three longitudinal stripes. Venter pale yellow with infuscations only on sternite VII. Metasomal segments I to V yellow to pale yellow, with several brown annular spots distally; segment V with better marked spots. Vesicle yellow without spots; aculeus yellow at the base and reddish at the tip. Chelicerae pale yellow with diffused variegated spots over their entire surface; better marked anteriorly; fingers pale yellow with reddish teeth. Pedipalps pale yellow, only slightly infuscate on the femur and patella; chela paler than patella; fingers pale yellow with the rows of granules slightly reddish. Legs yellow, densely marked with brownish spots. Carapace weakly granular to smooth; anterior margin almost straight; all furrows weak. Tergites weakly granular to smooth; median carina weak in all tergites. Pectines rather long; pectinal tooth count 17–18 in male. Sternites smooth with short semi-oval to round spiracles; VII with a few granulations and vestigial carinae. Metasomal segments with 10-10-8-8-5 weakly crenulate carinae; intercarinal spaces weakly granular to smooth. Telson with a fusiform shape, smooth with one vestigial ventral carina; aculeus moderately long and weakly curved; subaculear tubercle extremely reduced to vestigial. Pedipalps rather short; femur pentacarinate, with carinae weakly marked; patella with a few vestigial carinae; chela smooth; internal side of patella with some vestigial granules; all sides weakly granular, almost smooth; movable fingers with six almost linear rows of granules; two accessory granules present at the base of each row; extremity of movable fingers with three accessory granules. Leg tarsus with very numerous fine median setae ventrally; tibial spurs weakly developed on legs III and IV.

**Figure 3.**
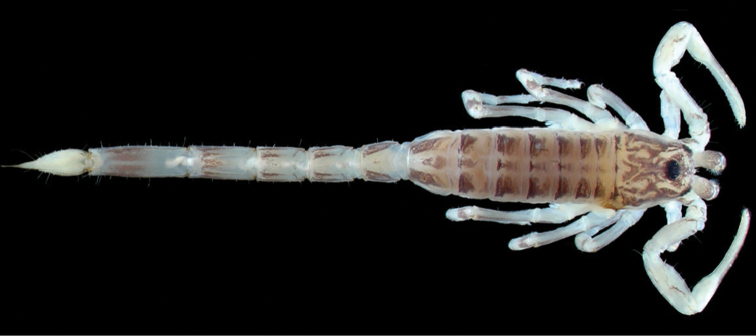
*Ananteris intermedia*, male holotype from St. Jean du Maroni (photo MNHN / E.-A. Leguin; 2012 Elsevier Masson SAS)

##### Ananteris kalina sp. n.

Taxon classificationAnimaliaScorpionesButhidae

http://zoobank.org/B08E25E5-D903-4C47-94F8-508E5BE467EF

Figs 4 , 5

###### Type material.

French Guiana, Mana, path of the Forêt des Sables Blancs, one male (holotype), deposited in the MNHN, J. Chevalier coll., 13/I/2018. French Guiana, Mana, path of the Forêt des Sables Blancs, one male (paratype), deposited in the EYPC, EY0107, J. Chevalier & B. Tan coll., 08/VII/2017.

###### Etymology.

The specific name refers to the ethnic group Kali’na, living in the area where the new species was found.

###### Diagnosis.

Total length 18.9 mm for male holotype (see morphometric values after the description). General coloration dark yellow, intensely marked with brownish variegated spots. Chelicerae pale yellow with variegated dark brown spots over the entire surface. Fingers with six rows of granules. Pectines of males holotype and paratype rather long with 16–17 and 17–17 teeth, respectively; female unknown. Telson with a fusiform shape and strong and spinoid subaculear tubercle. Carinae and granulation moderately to strongly marked. Metasomal segments with 10-8-8-8-5 weakly crenulate carina. Trichobothriotaxy, type A–β.

###### Description based on male holotype.

**Coloration.** Generally dark yellow with brown to dark brown variegated pigmented zones on the carapace, the tergites, and the appendages. Carapace dark yellow with dark brown spots on anterior, lateral and posterior edges; eyes surrounded by black pigment. Mesosoma dark yellow with confluent brown to dark brown zones forming three longitudinal stripes, one brownish surrounded by two reddish yellow ones. Venter yellow to pale yellow; coxapophysis and sternites with light brown zones on lateral edges. Metasomal segments I to V dark yellow with brown to dark brown variegated pigmented zones. Vesicle reddish yellow marbled with light brown zones; base of aculeus yellow, tip reddish. Chelicerae pale yellow with variegated dark brown spots over the entire surface; fingers yellowish with dark brown spots; teeth reddish yellow. Pedipalps yellowish, all segments almost entirely covered with brownish spots, dark brown on dorsal side and light brown on ventral side; tip of fingers pale yellow. Legs yellowish, intensely marked with brown to dark brown spots.

###### Morphology.

Carapace with moderately to strongly marked granulation; anterior margin almost straight, with a small median concavity; anterior median superciliary and posterior median carinae weak or absent; all furrows moderate to weak; median ocular tubercle distinctly anterior to the center of carapace; median eyes separated by approximately half of one ocular diameter; three pairs of reduced lateral eyes. Tergites with moderately to strongly marked granulation, similar to that of carapace; median carina moderately to weakly marked on all tergites, better marked posteriorly; tergite VII pentacarinate. Sternum subpentagonal. Pectines rather longs; pectinal tooth count 16–17 in male holotype (17–17 in male paratype); basal middle lamellae of pectines not dilated; fulcra absent. Sternites almost smooth, only VI and VII slightly granular; spiracles rather short, semi-oval; setation moderate; sternite VII with vestigial carinae; genital operculum divided longitudinally, each plate more or less suboval in shape. Metasomal segments with 10-8-8-8-5 weakly crenulate carinae; intercarinal spaces moderately to weakly granular; segment V slightly rounded and smooth. Telson with a fusiform shape, smooth; aculeus moderately long and weakly curved; subaculear tooth strong and spinoid. Pedipalp femur pentacarinate; patella and chela with weak to vestigial carinae, internal face of patella with some vestigial spinoid granules, all faces weakly granular, almost smooth; fixed and movable fingers with six rows of granules, two small external and one internal accessory granule present at the base of each row, three granules at the extremity of the fingers. Leg tarsus with very numerous, fine, median setae ventrally; tibial spurs strongly developed on legs III and IV. Cheliceral dentition characteristic of family Buthidae (Vachon, 1963); fixed finger with two strong basal teeth; movable finger with two vestigial basal teeth; ventral surface of both finger and manus with long, dense setae. Trichobothriotaxy of type A–β (Vachon, 1974). Morphometric values (in mm) of the male holotype. Total length including telson, 18.9. Carapace: length, 2.2; anterior width, 1.1; posterior width, 2.1. Mesosoma length, 5.1. Metasomal segments. I: length, 1.0; width, 1.2; II: length, 1.3; width, 1.1; III: length, 1.2; width, 1.1; IV: length, 1.8; width, 1.1; V: length, 3.1; width, 1.3; depth, 1.1. Telson: length, 3.2; width, 0.5; depth, 0.5. Pedipalp: femur length, 2.1, width, 0.4; patella length, 2.2, width, 0.5; chela length, 2.9, width, 0.3, depth, 0.3; movable finger length, 1.9.

**Figure 4.**
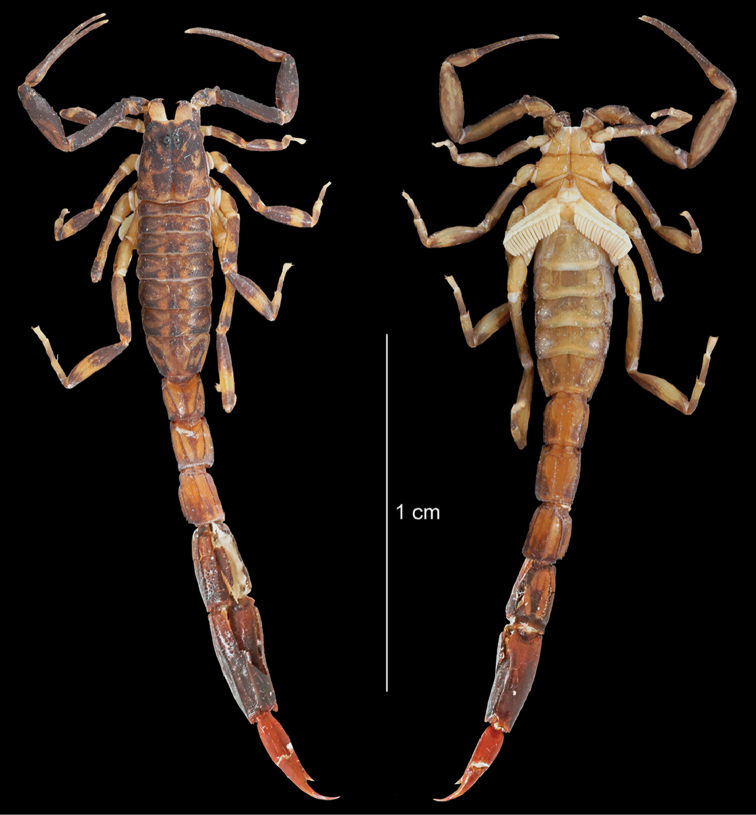
*Ananteris kalina* sp. n., male holotype from Mana. Habitus, dorsal and ventral aspect.

**Figure 5.**
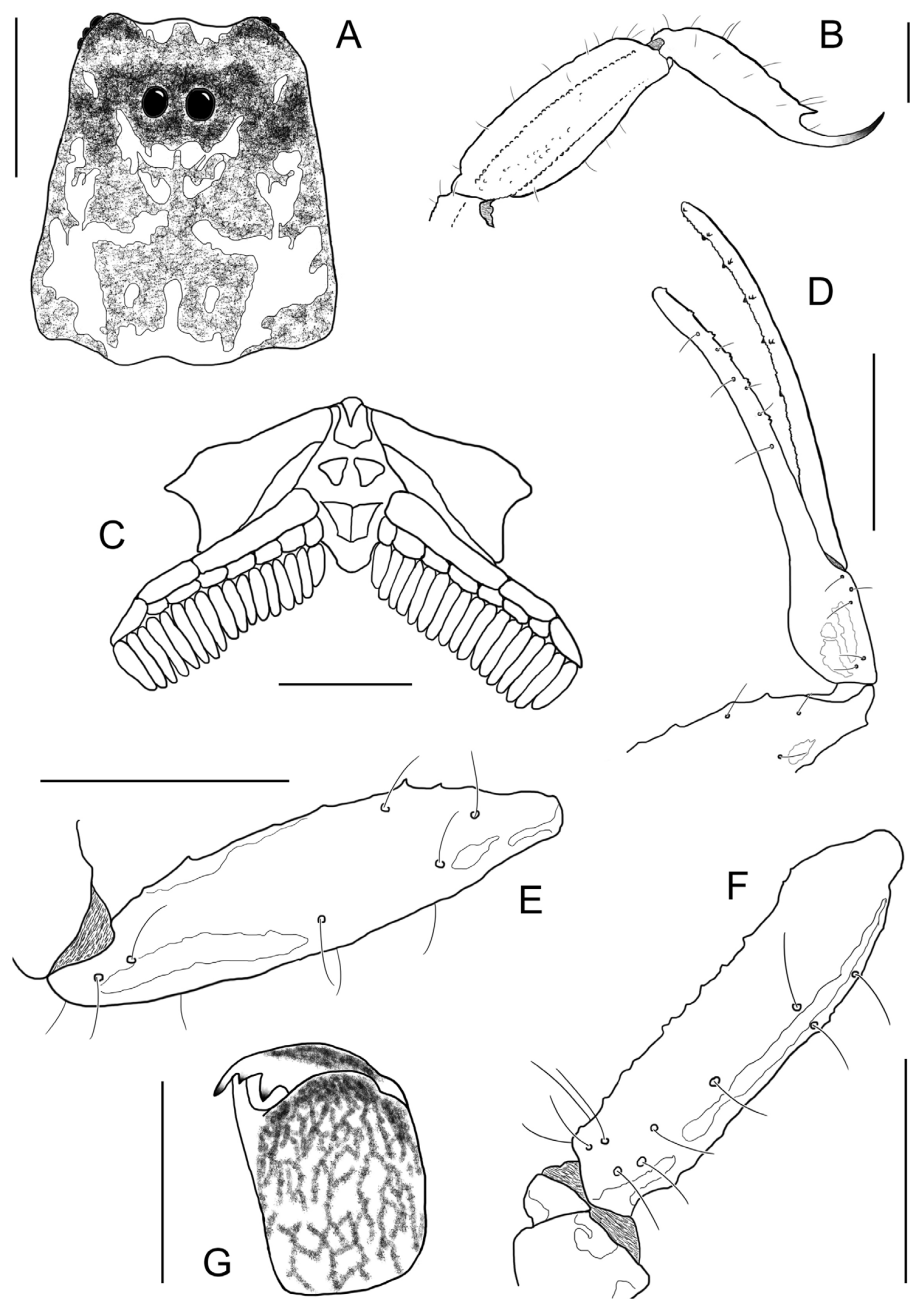
*Ananteris kalina* sp. n. male holotype. **A** Carapace **B** Metasomal segment V and telson, lateral aspect **C** Sternum, genital operculum, and pectines **D** Chela, dorso-external aspect **E** Patella, dorsal aspect **F** Femur, dorsal aspect **G** Chelicera. Scale bars: 1 mm except chelicera (**G**) 0.5 mm.

###### Relationships.

*Ananteris kalina* sp. n. can be readily distinguished from other species of the genus *Ananteris* and, in particular, from the three species occurring in the northern part of French Guiana, by the following main features:

– *A. guyanensis* Lourenço & Monod, 1999 (described from Saint-Eugène (Petit-Saut) and also found in Saut Sabbat (Mana)): (i) different pigmentation pattern on pedipalps and legs, (ii) chelicerae with variegated dark brown spots over the entire surface (uniformly yellow in *A. guyanensis*), (iii) metasomal segments with 10-8-8-8-5 carinae (10-10-10-8-5 in *A. guyanensis*).

– *A. intermedia* Lourenço, 2012 (described from Saint Jean du Maroni): (i) larger size (9.3 mm in total length for *A. intermedia*), (ii) subaculear tooth strong and spinoid (extremely reduced to vestigial in *A. intermedia*), (iii) metasomal segments with 10-8-8-8-5 carinae (10-10-8-8-5 in *A. intermedia*).

– *A. elisabethae* Lourenço, 2012 (described from Kourou): (i) darker general coloration (no spots or pigmented zones in *A. elisabethae*), (ii) chelicerae with variegated dark brown spots over the entire surface (uniformly yellow in *A. elisabethae*).

– The biotope where the new species occurs (coastal white-sand dry forest) is also different from biotope where other species of the genus are found in French Guiana (moist rainforest). The new species may be a possible endemic element of the white-sand coastal dry forest of Mana, French Guiana.

##### Ananteris polleti

Taxon classificationAnimaliaScorpionesButhidae

Lourenço, 2016

Fig. 6

###### References.

Lourenço 2016b.

###### Material.

Mitaraka massif, 433 m, tropical moist forest, in plateau, one male (holotype), deposited in the MNHN, MNHN/PNI Guyane 2015 (APA 973-1), M. Pollet coll., 2–8/III/2015. Mitaraka massif, 352 m, tropical moist forest, in slope, one male (paratype), deposited in the MNHN, MNHN/PNI Guyane 2015 (APA 973-1), M. Pollet coll., 25/II/2015–3/III/2015.

###### Diagnosis.

Species of small size compared to the average size of the other species within the genus (14.7 mm in total length for male holotype). General coloration yellow to brownish yellow with brown to dark brown variegated pigmented zones on the carapace, the tergites, and the appendages. Carapace yellow with dark brown spots on anterior, lateral and posterior edges; eyes surrounded by black pigment. Mesosoma yellow with confluent brownish zones on posterior and lateral edges of tergites. Venter yellow to pale yellow; coxapophysis and sternites infuscate. Metasomal segments I to V yellow; all segments marked with diffused brown spots. Vesicle yellow marbled with light brown zones; base of aculeus yellow, tip reddish. Chelicerae yellow with variegated blackish spots over the entire surface; fingers with blackish spots; teeth yellow. Pedipalps yellow; femur, patella, and chela strongly marked with dark brown spots; chela hand and fingers dark brown. Legs yellow, intensely marked with dark brown spots. Carapace with moderately to strongly marked granulation; anterior margin almost straight; all furrows moderate to weak. Tergites with moderately to strongly marked granulation, similar to that of carapace; median carina moderately to weakly marked on all tergites. Pectines small; pectinal tooth count 11–11 to 12–12 in male. Sternites smooth; only VII slightly granular; spiracles rather short; setation moderate; sternite VII with very weakly marked carinae and granulation. Metasomal segments I to III with ten crenulate carinae; segment IV with eight crenulate carinae; segment V slightly rounded and smooth, with vestigial carinae; intercarinal spaces moderately granular on all segments; dorsal and latero-dorsal carinae on segments II to IV with 3–4 posterior spinoid granules. Telson elongate and smooth; aculeus short and weakly curved; subaculear tooth strongly marked and spinoid. Pedipalps moderately short; femur pentacarinate; patella and chela with weak to vestigial carinae; internal side of patella with only vestigial spinoid granules; all sides weakly granular, almost smooth; fixed and movable fingers with six, almost linear, rows of granules; two small external and one internal accessory granule present at the base of each row; three granules at the extremity of the fingers. Leg tarsus with very numerous, fine, median setae ventrally; tibial spurs strongly developed on legs III and IV.

**Figure 6.**
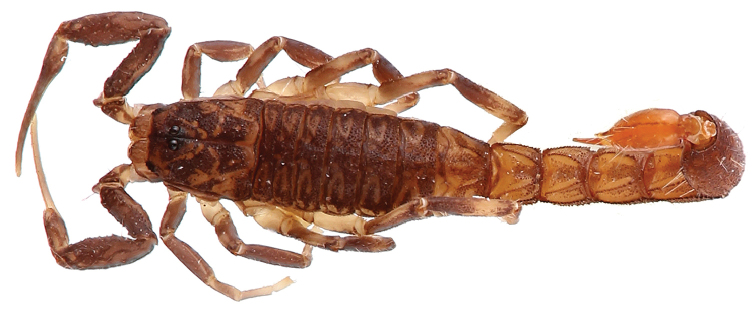
*Ananteris polleti*, male holotype from Mitaraka (photo MNHN / E.-A. Leguin; 2016 Elsevier Masson SAS).

##### Ananteris sabineae

Taxon classificationAnimaliaScorpionesButhidae

Lourenço, 2001

Fig. 7

###### References.

Lourenço 2001b, Lourenço 2003a, Lourenço 2016b.

###### Material.

Upper Ouarimapan river, trailhead camp of sentier indien, one female (holotype), MNHNRS6272, J. P. Gasc coll., VII/1972. Mitaraka Massif, layon D, in slope, winkler, one female, deposited in the MNHN, MNHN/PNI Guyane 2015 (MI15-0237-36), J. Orivel & F. PetitClerc coll., 23/II/2015–11/III/2015.

###### Diagnosis.

Species of medium size when compared with the average size of the other species of the genus (27.7 mm in total length for female holotype). General coloration basically brownish yellow, symmetrically marbled with dark reddish brown, producing an overall spotted appearance. Carapace dark yellow, almost totally covered with brown spots; eyes surrounded by black pigment. Mesosoma yellowish brown with confluent brown stripes and two diffused longitudinal yellowish stripes. Venter yellowish; sternite VII reddish yellow. Metasomal segments I to IV reddish yellow, with a few brown spots; segment V reddish brown, with less marked spots. Vesicle reddish yellow without spots. Chelicerae yellowish without any spots over their entire surface; fingers reddish brown. Pedipalps yellowish with a few diffused spots better marked on the femur; chelae darker than patella; fingers yellowish with the rows of granules slightly reddish. Legs yellowish with diffused spots, better marked than on pedipalps. Carapace moderately granular; anterior margin with a slight median concavity; all furrows moderate to feeble. Tergites moderately granular; median carina moderate in all tergites. Pectinal tooth count 19–18 in female. Sternites smooth with moderately elongate stigmata; VII granulated with vestigial carinae. Metasomal segments with 10-8-8-8-5 crenulate carinae; intercarinal spaces moderately to weakly granular. Telson moderately granular with three ventral carinae and with a fairly short and moderately curved aculeus; subaculear tooth strong and spinoid. Pedipalp femur pentacarinate; patella and chelae with a few vestigial carinae; internal side of patella with eight to nine spinoid granules; all sides feebly granular, almost smooth; movable fingers with seven oblique rows of granules; two accessory granules present at the base of each row. Leg tarsus with very numerous fine median setae ventrally; tibial spurs strongly developed on legs III and IV.

**Figure 7.**
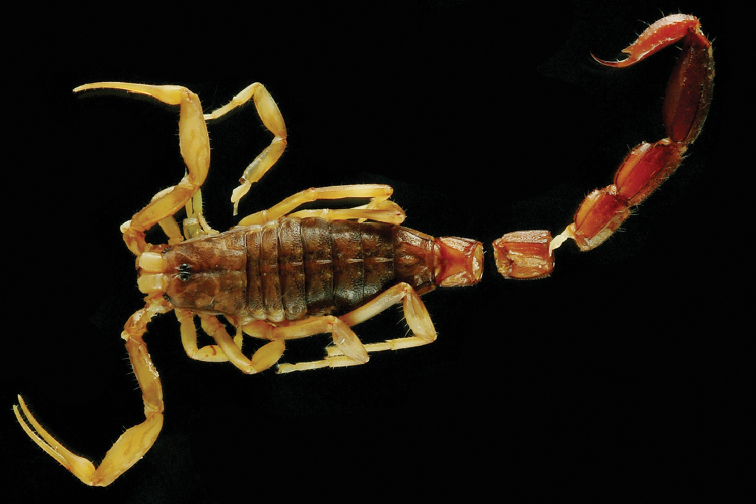
*Ananteris sabineae*, female holotype from upper Ouarimapan river (photo MNHN / E.-A. Leguin).

#### Genus *Isometrus* Hemprich & Ehrenberg, 1828

##### Isometrus maculatus

Taxon classificationAnimaliaScorpionesButhidae

(DeGeer, 1778)

Fig. 8

###### References.

DeGeer 1778, Lourenço 1983, Fet et al. 2000.

###### Material.

Cayenne, four males and three females, MNHN-RS-0899, M. Richard coll. Cayenne, one male and two females, MNHN-RS-3315, E. Abonnenc coll. Charvein-Maroni river, one male, MNHN-RS-3323, F. Geay coll., 1903. Coswine river, under rotten wood, one female, MNHN-RS-8299, J. Fretey coll., 16/V/1977. Morne-Cépéron, one male, MNHN RS3324, F. Geay coll., 1902. St. Jean du Maroni, two males, three females and two immatures, MNHN-RS3322, R. Benoist coll., 1914. St. Jean du Maroni, one male, MNHNRS7286, F. Geay coll., 1903. Disputed area between Oyapock and Amapa, one female, MNHN-RS0925. D. Villecourt coll., 1899. Disputed area between Oyapock and Amapa, one male, MNHNRS0914, F. Geay coll., 1899. Disputed area between Oyapock and Amapa, five males, sic females and one immature, MNHNRS0893, Lafon coll., 1872.

###### Diagnosis.

Species of medium to large size when compared with the average size of the other species of the genus, ranging from 50.1 mm (female) to 61.2 mm (male) in total length. General coloration yellowish to pale yellow, symmetrically marbled with blackish brown spots in both adults and juveniles. Carapace yellowish with blackish brown patterns; eyes surrounded by black pigment. Mesosoma yellowish with symmetrical blackish brown stripes. Venter yellowish; sternites III-VII with symmetrical brown spots. Metasomal segments pale yellow, with some diffuse, brownish spots. Vesicle pale yellow with basis of aculeus yellowish and tip of aculeus reddish brown. Chelicerae pale yellow with brownish variegated spots; base of fingers pale yellow, rest of fingers blackish brown, teeth reddish. Pedipalps pale yellow with brownish spots; chela fingers reddish brown; rows of granules on dentate margins of fingers dark reddish. Legs yellowish with diffuse spots. Carapace coarsely granular with a few smooth patches; anterior margin strongly emarginated, with an open V-shaped angle; carinae weakly developed. Tergites moderately granular; median carinae weak to moderate on I-VI, tergite VII with two lateral pairs of carinae moderate to strong. Pectinal tooth count ranging from 16–19 in male and 17–19 in female. Sternites smooth and shiny, VII with four granular carinae. Metasomal segments with 10-10-8-8-5 crenulate carinae; intercarinal spaces very weakly granular to smooth. Telson very weakly granular, almost smooth, with one vestigial ventral carina; subaculear tubercle marked and triangular, with two granules on the ventral surface. Pedipalp femur with all carinae crenulate; patella with seven crenulate carinae; chela with vestigial carinae; dentate margins of fixed and movable fingers with six linear rows of granules. Leg tibia with few setae, without spurs; basitarsus with some setae and two lateral pedal spurs; tarsus ventrally with two rows of short setae.

**Figure 8.**
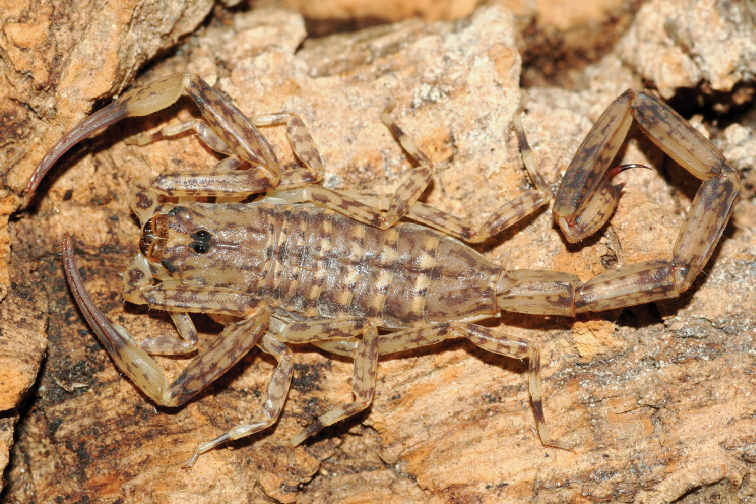
*Isometrus maculatus*, female from St. Jean du Maroni.

#### Genus *Jaguajir* Esposito, Yamaguti, Souza, Pinto da Rocha & Prendini, 2017

##### Jaguajir pintoi kourouensis

Taxon classificationAnimaliaScorpionesButhidae

(Lourenço, 2008)

Fig. 9

###### References.

Lourenço 1986, Lourenço and Pinto-da-Rocha 1997, Fet et al. 2000, Lourenço 2008, Esposito et al. 2017.

###### Material.

Region of Kourou, forest patches of Degrad path, one male (holotype), MNHNRS8631, mission M. Boulard & P. Pompanon coll., VIII/1975.

###### Diagnosis.

Large scorpion in relation to the species of the genus, with 89.7 mm in total length for the male holotype. Very dark coloration, uniformly blackish. Carapace and mesosoma blackish. Venter dark reddish to blackish. Metasomal segments I to V blackish. Vesicle dark reddish to blackish. Chelicerae dark reddish with a blackish thread; fingers dark. Pedipalps blackish; fingers reddish. Legs dark reddish, intensely spotted with blackish. Carapace strongly granular; anterior margin with a median concavity; anterior median and posterior median carinae strong; all furrows moderately deep. Tergites strongly granular; median carina strong in all tergites; tergite VII pentacarinate. Sternum triangular. Pectinal tooth count 24–25 in male. Sternites smooth with elongate spiracles; VII with four carinae and some lateral granulations. Metasomal segments I and II with ten carinae; III and IV with eight carinae; V with five carinae; inframedian carinae complete on II. Telson weakly granular, with a long and strongly curved aculeus; dorsal surface smooth; ventral surface granular; subaculear tooth absent. Pedipalp femur pentacarinate; patella with seven carinae; chela with nine carinae; internal side of patella with spinoid granules; all sides moderately to weakly granular; a very intense chaetotaxy can be observed in all segments; fixed and movable fingers with 9–10 oblique rows of granules; internal and external accessory granules strongly marked. Leg tarsus ventrally with numerous short fine setae.

**Figure 9.**
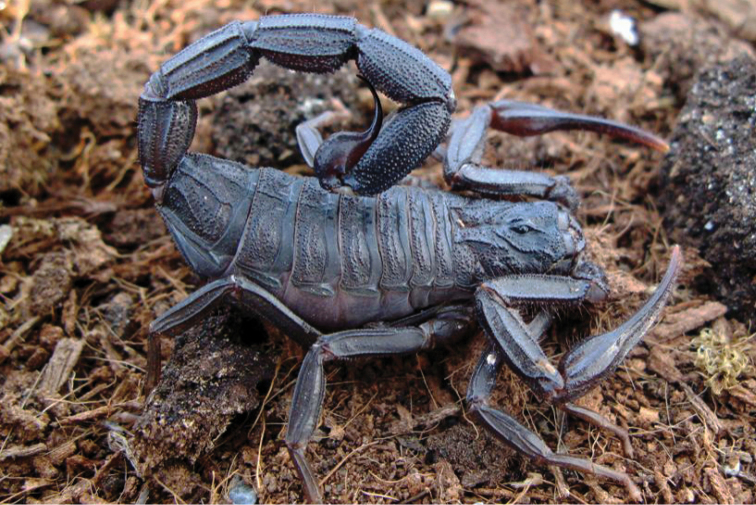
*Jaguajir pintoi kourouensis*, female from Kourou (photo G. Molisani).

#### Genus *Microananteris* Lourenço, 2003

##### Microananteris minor

Taxon classificationAnimaliaScorpionesButhidae

Lourenço, 2003

###### References.

Lourenço 2003b, Botero-Trujillo and Noriega 2011, Lourenço 2012c.

###### Material.

Central region, near the village of Saül, two km SW of the air field, dense humid forest at low altitude, in organic soil, extracted from Berlese method, one female (holotype), MNHNRS8602, J.M. Betsch leg., 19/III/1999.

###### Diagnosis.

Very small species when compared with the average size of most species of micro-buthid genera (11.7 mm in total length for female holotype). General coloration brownish yellow, symmetrically marbled with darker brown, producing an overall spotted appearance. Carapace yellowish, almost totally covered with brown spots; eyes surrounded by black pigment. Mesosoma yellowish brown with confluent brown stripes. Venter pale yellow. Metasomal segments I to V yellowish, with several pale brown spots; segment V with more marked spots. Vesicle yellowish with pale brownish spots laterally and ventrally; aculeus reddish. Chelicerae yellowish with variegated spots over their entire surface; more marked anteriorly; fingers yellowish with reddish teeth. Pedipalps yellowish densely marked with brownish spots better marked on the femur and patella; chela paler than patella; fingers brownish with the rows of granules slightly reddish. Legs yellowish, densely marked with brownish spots. Carapace moderately granular; anterior margin with a very slight median concavity, almost straight; all furrows moderate to weak. Tergites moderately to weakly granular; median carina moderate to weak in all tergites; tergite VII pentacarinate. Sternum subpentagonal. Pectines very small; pectinal tooth count 10–10 in female. Sternites smooth with short semi-oval spiracles; VII with a few granulations and vestigial carinae. Metasomal segments with 10-10-10-8-5 crenulate carinae; intercarinal spaces moderately to weakly granular. Telson with a pear-like shape, almost smooth with three ventral carinae; aculeus very short and moderately curved; subaculear tooth strong and almost rhomboid. Pedipalp femur pentacarinate; patella and chela with a few vestigial carinae; internal side of patella with some vestigial granules; all sides feebly granular, almost smooth; movable fingers with 6/7 almost linear rows of granules; two accessory granules present at the base of each row; extremity of movable fingers with three accessory granules. Leg tarsus with very numerous fine median setae ventrally; tibial spurs developed on leg IV but reduced on leg III.

#### Genus *Tityus* C. L. Koch, 1836

##### Tityus (Tityus) gasci

Taxon classificationAnimaliaScorpionesButhidae

Lourenço, 1981

Fig. 10

###### References.

Lourenço 1981, Lourenço 1983, Fet et al. 2000, Lourenço 2008.

###### Material.

Inini region, between Maripasoula and Antecume-Pata, one male (holotype), MNHN-RS7921, J.P. Gasc coll., VII-IX/1972, leg., 1975.

###### Diagnosis.

Species of medium size compared to the average size of the other species of the genus, with a total length of 63.4 mm for the male holotype. General coloration yellowish. Carapace yellowish with some shades of brownish; eyes surrounded with black pigment. Tergites yellowish with confluent pale yellow zones on tergites, making an incomplete rhomb on the tergite VII. Sternites dark yellow with a lighter triangular area on the posterior part of sternite V; sternum, genital operculum and pectines yellow ochre. Metasomal segments I to III yellowish, IV and V dark reddish. Vesicle dark reddish with basis of aculeus reddish yellow and tip of aculeus reddish black. Chelicerae yellowish with reddish teeth. Pedipalp femur and patella yellowish; chela reddish yellow with reddish fingers; base of movable finger dark, almost black. Legs yellowish with blackish pigment on carinae. Anterior margin of carapace moderately emarginated; carapace carinae weakly developed; anterior median carinae weak; intercarinal spaces weakly granular, almost smooth. Tergites almost smooth with only few granules on posterior area; tergites I-VI with one very weakly marked median carina; tergite VII pentacarinate with weakly marked carinae. Pectinal tooth count 17–18 in male; basal middle lamella not dilated. Sternites with spiracles almost linear. Metasomal segments with 10-10-8-8-5 weakly marked carinae; dorsal carinae of segments I to IV with one distal slightly spinoid granule; intercarinal spaces almost smooth. Telson with aculeus almost as long as the vesicle, strongly curved; subaculear tubercle well developed, with two dorsal teeth. Pedipalp femur pentacarinate; patella with seven carinae; chelae with nine carinae; dentate margins of fixed and movable fingers composed of 15–15 rows of granules.

**Figure 10.**
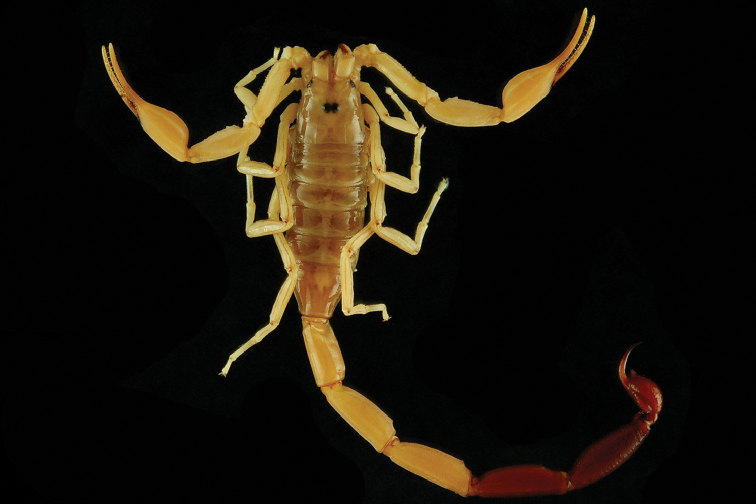
*Tityus gasci*, male holotype from Inini region (photo MNHN / E.-A. Leguin).

##### Tityus (Archaeotityus) mana

Taxon classificationAnimaliaScorpionesButhidae

Lourenço, 2012

Fig. 11

###### References.

Lourenço 1984, Lourenço 1992, Fet et al. 2000, Lourenço 2012d.

###### Material.

Path between Mana and Les Hattes, very sandy soil, one male (holotype), MNHNRS8084, M. Boulard & P. Pompanou coll., 8/VIII/1975. Mana, Organabo river, one female and one male, deposited in the RNA, J. Chevalier & Q. Uriot coll., 28/VII/2017. East Couachi, Organabo road, dry forest, one female (paratype), deposited in the MNHN, W. Lourenço coll., 22/VI/1987. Awala Yalimapo, Kanawa path, two males and one immature, deposited in the RNA, J. Chevalier coll., 03/VIII/2017. Awala Yalimapo, RNA path, three females and three immatures, deposited in the RNA, J. Chevalier coll., 01/IX/2017. Sinnamary, path of the Pripris de Yiyi, seven females and two immatures, deposited in the RNA, J. Chevalier & P. Gallier coll., 05/VIII/2017. Iracoubo, Savanne Grand Macoua, one female and one male, deposited in the RNA, J. Chevalier & Q. Uriot coll., 28/VII/2017. Iracoubo, sand quarry Moticase, one female and one male, deposited in the RNA, J. Chevalier & Q. Uriot coll., 28/VII/2017.

###### Diagnosis.

Small to moderate species when compared with the average size of the other species of the genus, ranging from 29.9 mm (male) to 38.5 mm (female) in total length. General coloration yellowish to pale yellow with only residual variegated pale brown spots over the body and appendages. Carapace yellowish with residual spots on the posterior and central zones; eyes surrounded with black pigment. Mesosoma yellowish with pale brown variegated spots on the posterior margins of tergites. Venter yellowish; sternites yellowish with pale brown variegated spots on the posterior margins; sternum, genital operculum and pectines pale yellow. Metasomal segments I to IV yellowish, V reddish yellow, with dark spots laterally and ventrally. Vesicle reddish to dark reddish; aculeus reddish. Chelicerae yellowish with variegated dark brown spots on the front part; fingers yellowish with dark brown spots at their basis; teeth reddish. Pedipalps yellowish with only some vestigial spots on the femur and patella of male. Legs yellowish with residual variegated spots on all segments. Anterior margin of carapace only moderately emarginated; carapace carinae weakly developed; all furrows weak; intercarinal spaces weakly granular. Tergites I-VI with one moderately marked median carina; tergite VII pentacarinate, lateral pairs of carinae moderately marked, median carinae marked only on proximal third; intercarinal spaces weakly granular. Pectines small with moderate fulcra; basal middle lamella not dilated in female; pectinal tooth count ranging from 16–17 in male and 14–17 in female. Sternites surface with a residual granulation, almost smooth; carinae absent on III-VI, four weak to moderate carinae on VII; spiracles slit-like but short. Metasomal segments with 10-10-8-8-5 carinae; dorsal carinae of segments I to IV with one strong distal spinoid granule, better marked in female; intercarinal spaces weakly granular. Telson smooth in males; with one ventral and four vestigial lateral carinae in the female; aculeus shorter than vesicle, moderately curved; subaculear tubercle short and strongly rhomboid, with two dorsal teeth. Pedipalp femur pentacarinate; all carinae moderately to strongly crenulate; patella with seven carinae; internal carina with strong spinoid granules; chelae with 8–9 strongly marked carinae; all sides weakly granular; carinae and granules better marked in female; dentate margins of fixed and movable fingers composed of 13–14 oblique rows of granules. Ventral aspect of leg tarsi with numerous thin setae; tibial spurs absent; pedal spurs present but vestigial in all legs.

**Figure 11.**
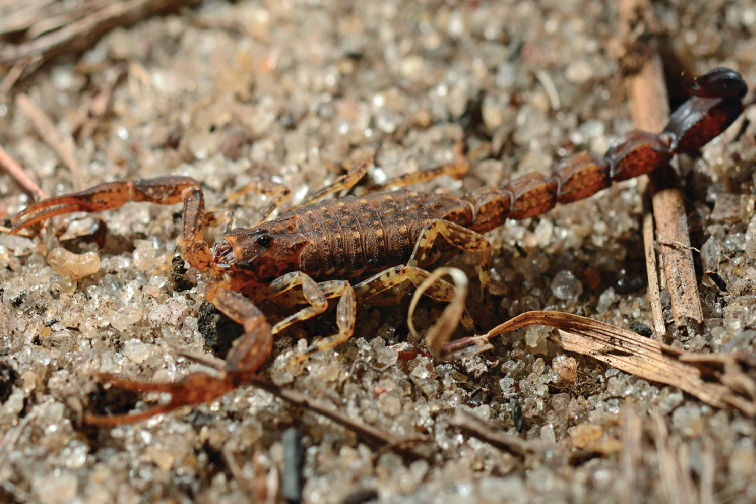
*Tityus mana*, female from Awala Yalimapo (photo J. Chevalier).

##### Tityus (Atreus) obscurus

Taxon classificationAnimaliaScorpionesButhidae

(Gervais, 1843)

Figs 12 , 13

###### References.

Gervais 1843, Pocock 1897, Lourenço 1983, Lourenço 1997b, Fet et al. 2000, Lourenço 2002a, Lourenço and Leguin 2008, Stockmann and Ythier 2010.

###### Material.

Cayenne, one adult female (lectotype) and one immature female (paralectotype), MNHN-RS3298, Mr. Leschenault & Mr. Doumerc coll. Cayenne, one female, MNHNRS-0855, Noirot coll., 1890. Cayenne, one male and one female, MNHNRS0861, M. Melinon coll., 1877. Cayenne, three females, MNHNRS3297, M. Melinon coll., 1876. Cayenne, two females, MNHNRS3299, R. Pinchon leg., 1953. Cayenne, three males, MNHNRS3314, E. Abonnenc coll., two males, MNHNRS3317, E. Abonnenc coll., Inst. Pasteur leg. Cayenne, Montabo, in forest, one female, MNHNRS3319, III/1949. Cayenne, caught from a cat in a house, one female, MNHN-RS3321, D. Destombes coll., VI/1950. Cayenne, one female, MNHNRS3325, F. Geay coll., 1902. Cayenne, one female, MNHN-RS-0846, St. Laurent coll., 1899. Border with Para, one male and one female, MNHNRS-3286, 1900. Cayenne, Mt. St. Martin, forest, one female, MNHNRS7926, D. Quintero coll., 10/XII/1972. Cayenne, one male, MNHNRS8081, M. Condamin leg., 25/VI/1976. Cayenne, Mont Bourda, one immature, deposited in the RNA, J. Chevalier & B. Tan coll., 09/VII/2017. Cayenne region, one male, deposited in the MHNG, Freitag leg., IX/1987. AmaroneBaraquin, one male and one female, MNHNRS0849, 1900. AntecumePata, in forest, one female, MNHN-RS-6268, J.P. Gasc coll., 18/VII/1972. AntecumePata, in forest, one female, MNHN-RS-6269, J.P. Gasc coll., 18/VII/1972. Antecume-Pata, one female, MNHNRS7918, J.P. Gasc leg., 1975. Antecume-Pata, one male, MNHN-RS7919, J.P. Gasc leg., 1975. Antecume-Pata, one female, MNHN-RS-7920, J.P. Gasc leg., 1975. Downstream from Saut Pararé on Arataye river, Approuague tributary, two males and one female, MNHNRS7389, J.P. Gasc coll., IV-V/1979. Downstream from Saut Pararé on Arataye river, Approuague tributary, one male, MNHNRS7391, J.P. Gasc coll., IVV/1979. Camopi, Oyapock valley, one male and one female, MNHNRS3393, mission E. Aubert de la Rüe coll., 18/XII/1948. Upper Approuage, in forest, one male, MNHNRS3301, III/1946. Upper Oyapock, between Mount Orière and Dégrad Galoupa, two females, MNHNRS3310, mission E. Aubert de la Rüe coll., 1948–49. Kaw, one female, MNHNRS8296, J. Lescure coll., 2730/IV/1977. Mitaraka Massif, hand catch, one male, two females and one immature female, deposited in the MNHN, MNHN/PNI Guyane 2015, E. Poirier, P.H. Dalens & J. Touroult coll., 11–18/III/2015. Mitaraka Massif, camp, layon D, tropical moist forest, in plateau, winkler, two females, deposited in the MNHN, MNHN/PNI Guyane 2015, J. Orivel & F. PetitClerc coll., 23/II/2015–11/III/2015. Apatou, Crevette river, one male, one female and one immature male, deposited in the RNA, J. Chevalier & P. Gallier coll., 30/VI/2017. Sinnamary, path of the Canceler river, under a palm tree’s bark, one female and one male, deposited in the RNA, J. Chevalier & P. Gallier coll., 05/VIII/2017. Iracoubo, Savanne Grand Macoua, one female, deposited in the RNA, J. Chevalier & Q. Uriot coll., 28/VII/2017. Mounts of Montsinery, one female, MNHNRS5252, F. Geay coll., II/1902. Oyapock, two males, MNHN-RS0845, F. Geay coll., 1900. Oyapock, one female, MNHNRS0862, F. Geay coll., 1900. Oyapock, one male, MNHN-RS3305, mission E. Aubert de la Rüe coll., 1/XII/1948. Saül, one male and three females, MNHNRS5286, Balachowsky leg., 27/X/1969. Saül, Gros Arbres trail, one female, deposited in the RNA, J. Chevalier coll., 23/VIII/2017. Saül, Belvédère, one juvenile, deposited in the RNA, J. Chevalier coll., 21–22/VIII/2017. Saut Sabbat, one female, MNHNRS8208, D. Kopp coll., 8/VII/1976. Saut Sabbat, one immature female, deposited in the MNHN, E. Ythier coll., 03–11/XI/2010. St. Jean du Maroni, one female, MNHNRS0856, R. Benoist coll., 1914. Yanioué, upper Oyapock, upper Camopi, upstream waterfall, one male, MNHNRS3295, mission E. Aubert de la Rüe coll., II/1949. Cacao, four males and one immature, deposited in the MHNG, Chippaux leg., X/1983. Cacao, one male, deposited in the MHNG, W. Lourenço leg., II/1989. Cacao, one male, deposited in the MHNG, P. Soler leg., I/1992. Kourou, one male, deposited in the MHNG, R. Garrouste leg., 16/II/1995. Kourou, one male, deposited in the MHNG, Freitag leg., 9/X/1987. Petit-Saut, in canopy with radeau des cimes, one immature female, deposited in the MHNG, H.P. Aberlenc coll., 4/XI/1989. Trinité reserve, Aya river, pitfall trap, one immature female, deposited in the MNHN, C. Courtial coll., X/2009. Trinité reserve, Aya river, one immature male, deposited in the MNHN, C. Courtial coll., X/2008. Trinité reserve, Aya River, two males and one female, deposited in the MNHN, C. Courtial coll., XII/2010. Matoury, one male and one immature, deposited in the EYPC, EY0042, E. Ythier coll., 03–11/XI/2010. Salobroc, two immatures, MNHNRS0812. Guyane, three females, MNHN-RS0810, M. Lafon coll., 1872. Guyane, two males and one female, MNHNRS0844, G. Dewer coll., 1897. Guyane, one female, MNHN-RS0850, Viguier coll., 1877. Guyane, one female, MNHNRS8250, D. Kopp coll., 3/VII/1976. Between Oyapock and Amapa (disputed area), one female, MNHNRS0852, Villecourt coll., 1899. Between Oyapock and Mount SociatMarcel, on the ground, base camp, river bank, Eleuponsin, one female, MNHNRS8076, J. P. Gasc coll. III/1976. Paranama, one female, MNHNRS8251, D. Kopp coll., 3/VII/1976. Franco-brazilian region, two females, MNHNRS0839, F. Geay coll., 1899. Le Para (?), five males and five females, MNHN-RS0854.

###### Diagnosis.

Species of large size when compared with the average size of the other species within the genus, ranging from 75.7 to 100 mm in total length. General coloration uniformly dark brown to blackish. Carapace and mesosoma uniformly dark brown to blackish. Sternites with some pale zones; a triangular smooth testaceous area on the middle of the posterior border of the sternite III; pectines testaceous. Metasomal segments I to V and telson uniformly dark brown to blackish. In most cases the juvenile instars are yellowish or reddish yellow, with very numerous variegated spots, these spots being not visible in the adults as a result of the very marked sclerification of the cuticle. Chelicerae yellowish with variegated dark brown spots over the entire surface; fingers brownish; teeth dark reddish. Pedipalps dark reddish; chela fingers dark brown to blackish with tip yellowish. Legs yellowish almost entirely covered with brownish spots, except on telotarsus. Number of pectine teeth ranging from 18 to 22 teeth in both sexes; the basal middle lamellae strongly dilated in females. Metasomal segments I and II with ten carinae; segments III and IV with eight carinae; segment V with five carinae. Telson with a strong spinoid subaculear tooth present that can be moderate in size or almost totally absent in very large specimens. Dentate margins of pedipalp chela fixed and movable fingers with 15–17 oblique rows of granules. Very strong sexual dimorphism; male pedipalps are longer and more slender than those of the females; metasoma of the male is also longer than the one of the female.

**Figure 12.**
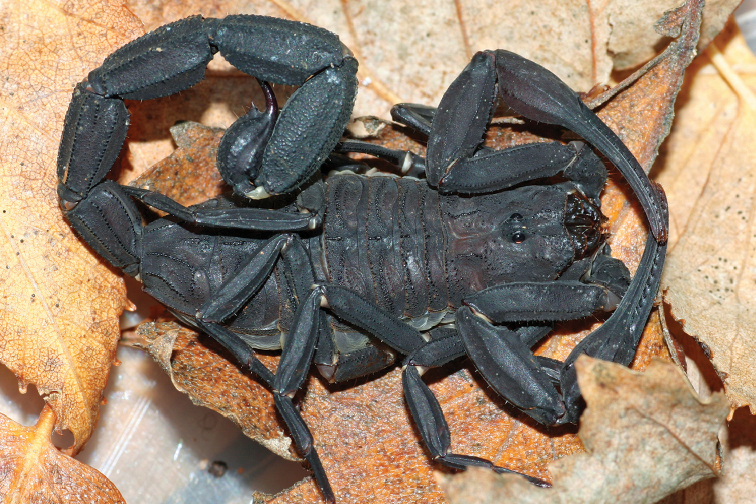
*Tityus obscurus*, adult female from Cayenne.

**Figure 13.**
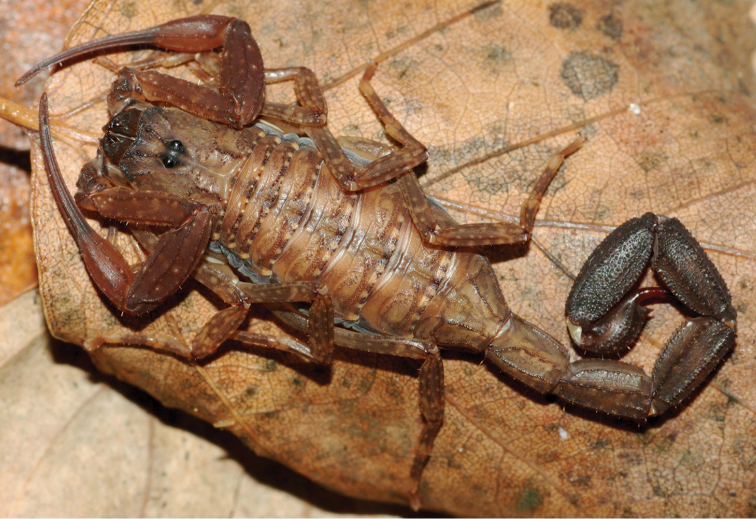
*Tityus obscurus*, juvenile from Saut Sabbat.

##### Tityus (Archaeotityus) silvestris

Taxon classificationAnimaliaScorpionesButhidae

Pocock, 1897

Fig. 14

###### References.

Pocock 1897, Lourenço 1983, Lourenço 1984, Lourenço 1988a, Lourenço 1992, Lourenço 1997b, Fet et al. 2000, Lourenço 2002a, Lourenço 2008, Stockmann and Ythier 2010.

###### Material.

Downstream from Saut Pararé on Arataye River, Approuague tributary, one male, MNHNRS7390.1, J.P. Gasc coll., IV-X/1979. Ilet la Mère, one female, MNHNRS8298, J. Lescure coll., 25/VII/1977. St. Jean du Maroni, one female, MNHNRS3322, R. Benoist coll., 1914. Cacao, one male, deposited in the MHNG, Chippaux coll., X/1983. Mitaraka Massif, 433 m, tropical moist forest, in plateau, one male, deposited in the MNHN, MNHN/PNI Guyane 2015 (APA 973-1), M. Pollet coll., 2–8/III/2015. Mitaraka Massif, pointe Macaria, hand catch, one female, deposited in the MNHN, MNHN/PNI Guyane 2015 (APA 973-1), E. Poirier, P.H. Dalens & J. Touroult coll., 24–27/II/2015. Saül, Popote Kanawa, one immature, deposited in the RNA, J. Chevalier coll., 24/VIII/2017. Cayenne, Mont Bourda, two females and six immatures, deposited in the RNA, J. Chevalier coll., 09/VII/2017. Disputed area between Oyapock and Amapa, one female, MNHNRS0820, D. Villecourt coll., 1899.

###### Diagnosis.

Small to moderate species when compared with the average size of the other species of the genus, with a very variable size ranging from 25 to 45 mm in total length. General coloration yellowish to pale yellow densely spotted with brownish to blackish pigmentation. Carapace yellowish with brownish yellow pigmentation and brownish spots; a conspicuous bright yellow T-shaped mark on the anterior part. Tergites yellowish with brownish yellow pigmentation and brownish spots. Sternites yellowish with brownish spots. Metasomal segments I-III yellowish with brownish spots, IV and V reddish yellow. Vesicle reddish brown; aculeus reddish brown at the base and blackish at the tip. Pedipalps yellowish with brownish spots; chela fingers reddish yellow. Legs yellowish with brownish spots. This species presents a very complex pattern of polymorphism. Number of pectine teeth ranging from 14 to 16 in male and 12 to 15 in female; basal middle lamellae of female pectines not dilated. Metasomal segments I to IV without any spinoid posterior granule; segments IV and V of males bigger than those of females. Telson with a strong and rhomboid subaculear tooth. Dentate margins of pedipalp fingers composed of 15–16 oblique rows of granules in both males and females; male patella and metasomal segments generally much more bulky than those of females.

**Figure 14.**
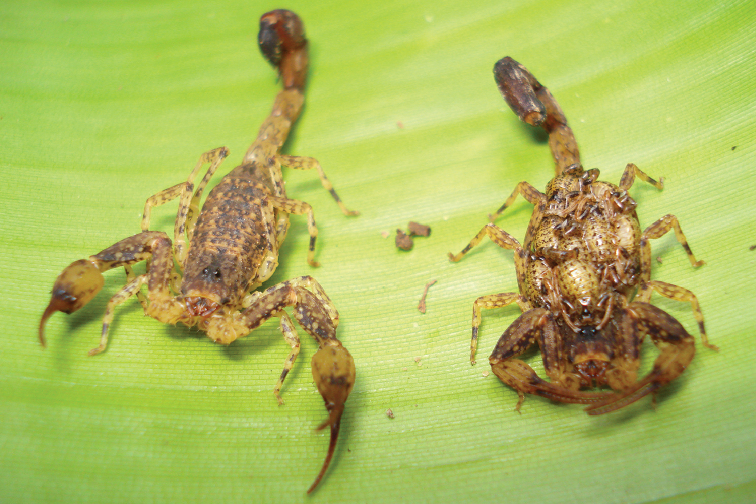
*Tityus silvestris*, male (left) and female with juveniles (right) from Saül (photo A. Thillien).

### Family CHACTIDAE Pocock, 1893

#### Genus *Auyantepuia* Gonzalez-Sponga, 1978

##### Auyantepuia aluku sp. n.

Taxon classificationAnimaliaScorpionesChactidae

http://zoobank.org/5A303DD8-21BA-46BF-9F85-F25C43991E42

Figs 15 , 16

###### Type material.

French Guiana, Apatou, Crevette river, one female (holotype), deposited in the MNHN, J. Chevalier & P. Gallier coll., 30/VI/2017. Apatou, Crevette river, four females (paratypes), deposited in the EYPC, EY0094, J. Chevalier & P. Gallier coll., 30/VI/2017.

###### Etymology.

The specific name refers to the ethnic group Aluku, living in the area where the new species was found.

###### Diagnosis.

Total length ranging from 20.4 to 21.5 mm (20.9 mm in total length for female holotype; see morphometric values after the description). Coloration reddish brown, with carapace, chelicerae, pedipalps and legs marked with darker spots. Tergites brownish with confluent reddish yellow spots, on the sides and the middle of tergites, forming a yellowish longitudinal median stripe. Posterior half of ventral side of segments I to IV yellowish, without spots. Body and appendages almost smooth, shiny; chela weakly granulated, dorso-internal carina inconspicuous; ventral posterior spinoid granulations on metasomal segment V. Pectines of female holotype and paratypes with 5–6 to 6–6 teeth; male unknown. Trichobothrial pattern of type C neobothriotaxic ‘majorante’.

###### Description based on female holotype.

**Coloration.** General coloration reddish brown. Carapace reddish yellow, marked with brownish variegated spots around the ocular tubercle and on the anterior and posterior edges of the carapace; ocular tubercle darker, almost black. Tergites brownish with confluent reddish yellow spots, on the sides and the middle of tergites, forming a yellowish longitudinal median stripe. Venter and sternites yellowish; sternum yellowish with darker spots on the middle and anterior edge; genital opercle yellowish; pectines pale yellow. Metasomal segments reddish yellow, marked with variegated brownish spots on lateral and dorsal sides of segments I to V and on ventral side of segments IV and V; posterior half of ventral side of segments I to IV yellowish, without spots. Vesicle reddish yellow with basis of aculeus blackish and tip of aculeus reddish. Chelicerae yellowish, with variegated dark brown spots; fingers reddish yellow; teeth reddish. Pedipalps reddish yellow, with longitudinal dark brown spots. Legs yellowish, intensely marked with brownish spots.

###### Morphology.

Carapace acarinate, shiny, and almost smooth, with only some minute granulations on lateral edges; furrows shallow; anterior edge emarginate. Tergites acarinate, shiny, and almost smooth. Sternum pentagonal, wider than long. Pectinal tooth count 6–6 in female holotype (5–6 to 6–6 in females paratypes), fulcra absent. Sternites smooth and shiny, VII acarinate; spiracles rounded in shape. Only metasomal segments IV and V longer than wide; metasomal tegument almost lustrous, without granulation, and with a few punctations; segment V with posterior spinoid granulation ventrally; carinae on segments I-V vestigial or absent; only dorso-lateral carinae are weakly marked on segments I to IV. Pedipalp femur with dorsal internal, dorsal external and ventral internal carinae moderately marked, internal face weakly granular, other faces smooth; patella smooth, with vestigial carinae; chela weakly granulated, almost smooth, with dorso-internal carina weakly marked; dentate margins on fixed and movable fingers with six rows of granules. Chelicerae with dentition typical of the family Chactidae (Vachon, 1963), and with dense setation ventrally and internally. Trichobothriotaxy of type C; neobothriotaxic ‘majorante’ (Vachon, 1974). Morphometric values (in mm) of the female holotype. Total length including telson, 20.9. Carapace: length, 3.2; anterior width, 1.9; posterior width, 3.2. Mesosoma length, 7.2. Metasomal segments. I: length, 1.3; width, 2.0; II: length, 1.3; width, 1.7; III: length, 1.4; width, 1.5; IV: length, 1.7; width, 1.4; V: length, 2.6; width, 1.2; depth, 1.2. Telson: length, 2.2; width, 1.1; depth, 0.8. Pedipalp: femur length, 1.5, width, 0.9; patella length, 2.1, width, 1.0; chela length, 4.6, width, 2.1, depth, 1.7; movable finger length, 2.3.

**Figure 15.**
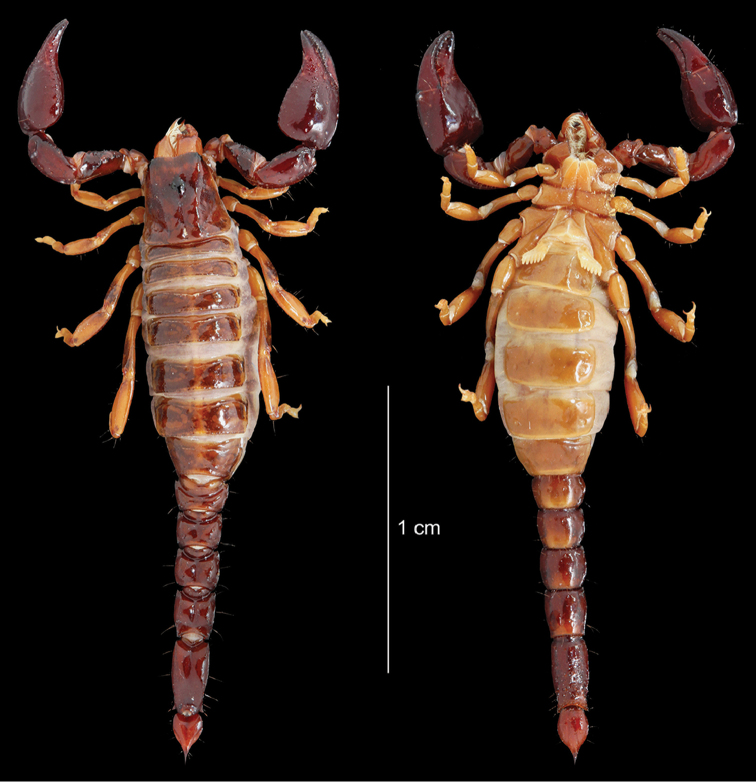
*Auyantepuia aluku* sp. n., female holotype from Apatou. Habitus, dorsal and ventral aspect.

**Figure 16.**
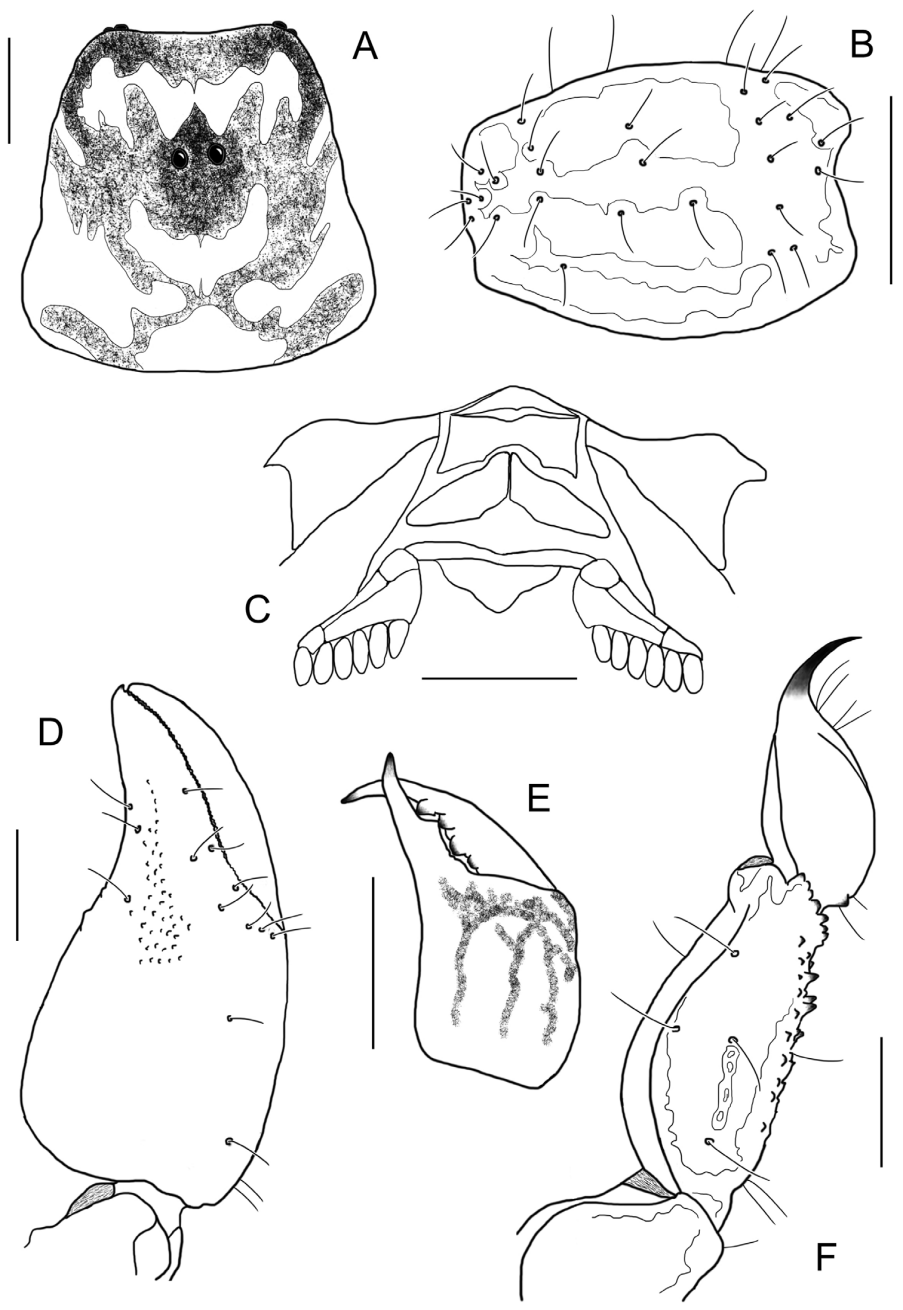
*Auyantepuia aluku* sp. n. female holotype. **A** Carapace **B** Patella, external aspect **C** Sternum, genital operculum and pectines **D** Chela, dorso-external aspect **E** Chelicera **F** Metasomal segment V and telson, lateral aspect. Scale bars: 1 mm except chelicera 0.5 mm (**E**).

###### Relationships.

*Auyantepuia aluku* sp. n. can be readily distinguished from other species of the genus *Auyantepuia* and, in particular, from the three species occurring in the northern part of French Guiana and Suriname, by the following main features:

– *A. laurae* Ythier, 2015 (described from Saut Sabbat, Mana): (i) smaller size (27.5 to 28.2 mm in total length for *A. laurae*), (ii) tergites with confluent reddish yellow spots forming a yellowish longitudinal median stripe (no stripe in *A. laurae*), (iii) posterior half of ventral side of segments I to IV yellowish, without spots (ventral side of segments I to III entirely yellowish, without spots in *A. laurae*).

– *A. gaillardi* Lourenço, 1983 (described from Saint-Laurent-du-Maroni): (i) smaller size (26.9 mm in total length for the female of *A. gaillardi*), (ii) carapace, tergites, chelicerae, pedipalps and legs marked with darker spots (uniform coloration without darker spots in *A. gaillardi*), (iii) posterior half of ventral side of segments I to IV yellowish, without spots (all segments uniformly reddish in *A. gaillardi*).

– *A. surinamensis* Lourenço & Duhem, 2010 (described from Albina/Moengo, Suriname): (i) tergites with confluent reddish yellow spots forming a yellowish longitudinal median stripe (no stripe in *A. surinamensis*), (ii) posterior half of ventral side of segments I to IV yellowish, without spots (all segments reddish uniformly and intensely marked with brownish spots in *A. surinamensis*).

##### Auyantepuia aurum sp. n.

Taxon classificationAnimaliaScorpionesChactidae

http://zoobank.org/71974C08-003E-4D34-9882-D4882268AF6B

Figs 17 , 18

###### Type material.

French Guiana, Saül, Gros arbres trail, one male (holotype), deposited in the MNHN, J. Chevalier, B. Tan & R. Legallic coll., 21–22/VIII/2017. Saül, Gros arbres trail, one male (paratype), deposited in the EYPC, EY0095, J. Chevalier, B. Tan & R. Legallic coll., 21–22/VIII/2017.

###### Etymology.

The specific name is allusive to gold (Latin *aurum*) panning, for which the village of Saül (where the new species occurs) was founded at the beginning of the 19^th^ century.

###### Diagnosis.

Total length 25.2 mm for male holotype and 28.1 mm for male paratype (see morphometric values after the description). Coloration reddish brown, with carapace, chelicerae, pedipalps and legs marked with darker spots. Tergites brownish with confluent yellowish spots, on the sides and the middle of tergites, forming a yellowish longitudinal median stripe. Ventral side of segments I and II yellowish, without spots. Body and appendages weakly to moderately granulated; chela moderately granulated, dorso-internal carina inconspicuous; granulations on lateral sides of all metasomal segments and on ventral side of segments III to V, spinoid on V. Pectines of males holotype and paratypes with 7–7 and 5–7 teeth, respectively; female unknown. Trichobothrial pattern of type C neobothriotaxic ‘majorante’.

###### Description based on male holotype.

**Coloration.** General coloration reddish brown. Carapace reddish yellow, marked with brownish variegated spots around the ocular tubercle and on the anterior and posterior edges of the carapace; ocular tubercle darker, almost black. Tergites brownish with confluent yellowish spots, on the sides and the middle of tergites, forming a yellowish longitudinal median stripe. Venter and sternites yellowish; sternum yellowish with darker spots on the middle and anterior edge; genital opercle yellowish; pectines pale yellow. Metasomal segments reddish yellow, marked with variegated brownish spots on lateral and dorsal sides of segments I to V and on ventral side of segments III, IV and V; ventral side of segments I and II yellowish, without spots. Vesicle reddish yellow with basis of aculeus blackish and tip of aculeus reddish. Chelicerae yellowish, with variegated dark brown spots; fingers reddish yellow with dark brown spots at their basis; teeth reddish. Pedipalps reddish yellow, with longitudinal dark brown spots. Legs yellowish, intensely marked with brownish spots.

###### Morphology.

Carapace acarinate, with some fine granulations on central, lateral and posterior parts; furrows shallow; anterior edge emarginate. Tergites acarinate, with some fine granulations, stronger on their posterior edges. Sternum pentagonal, wider than long. Pectinal tooth count 7–7 in male holotype (5–7 in male paratype), fulcra absent. Sternites smooth and shiny, VII acarinate; spiracles rounded in shape. Metasomal segments III, IV and V longer than wide; metasomal tegument with medium size granulation on lateral sides of all segments and on ventral side of segments III to V, spinoid on V; carinae on segments I-V vestigial, only dorso-lateral carinae are weakly marked on all segments. Pedipalp femur with dorsal internal, dorsal external and ventral internal carinae moderately marked, internal face weakly granular, other faces smooth; patella smooth, with vestigial carinae; chela moderately granulated, with dorso-internal carina weakly marked; dentate margins on fixed and movable fingers with six rows of granules. Chelicerae with dentition typical of the family Chactidae (Vachon, 1963), and with dense setation ventrally and internally. Trichobothriotaxy of type C; neobothriotaxic ‘majorante’ (Vachon, 1974). Morphometric values (in mm) of the male holotype. Total length including telson, 25.2. Carapace: length, 3.9; anterior width, 2.2; posterior width, 3.6. Mesosoma length, 7.7. Metasomal segments. I: length, 1.4; width, 2.0; II: length, 1.9; width, 2.0; III: length, 2.0; width, 1.7; IV: length, 2.3; width, 1.7; V: length, 3.3; width, 1.6; depth, 1.4. Telson: length, 2.7; width, 1.4; depth, 1.2. Pedipalp: femur length, 2.1, width, 1.0; patella length, 2.8, width, 1.1; chela length, 5.2, width, 2.3, depth, 1.8; movable finger length, 2.9.

**Figure 17.**
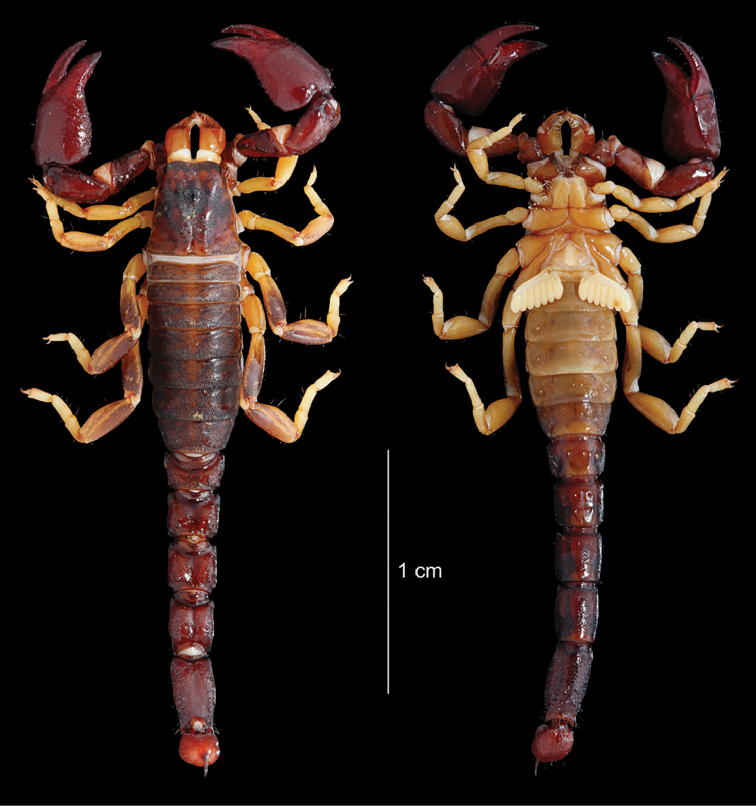
*Auyantepuia aurum* sp. n., male holotype from Saül. Habitus, dorsal and ventral aspect.

**Figure 18.**
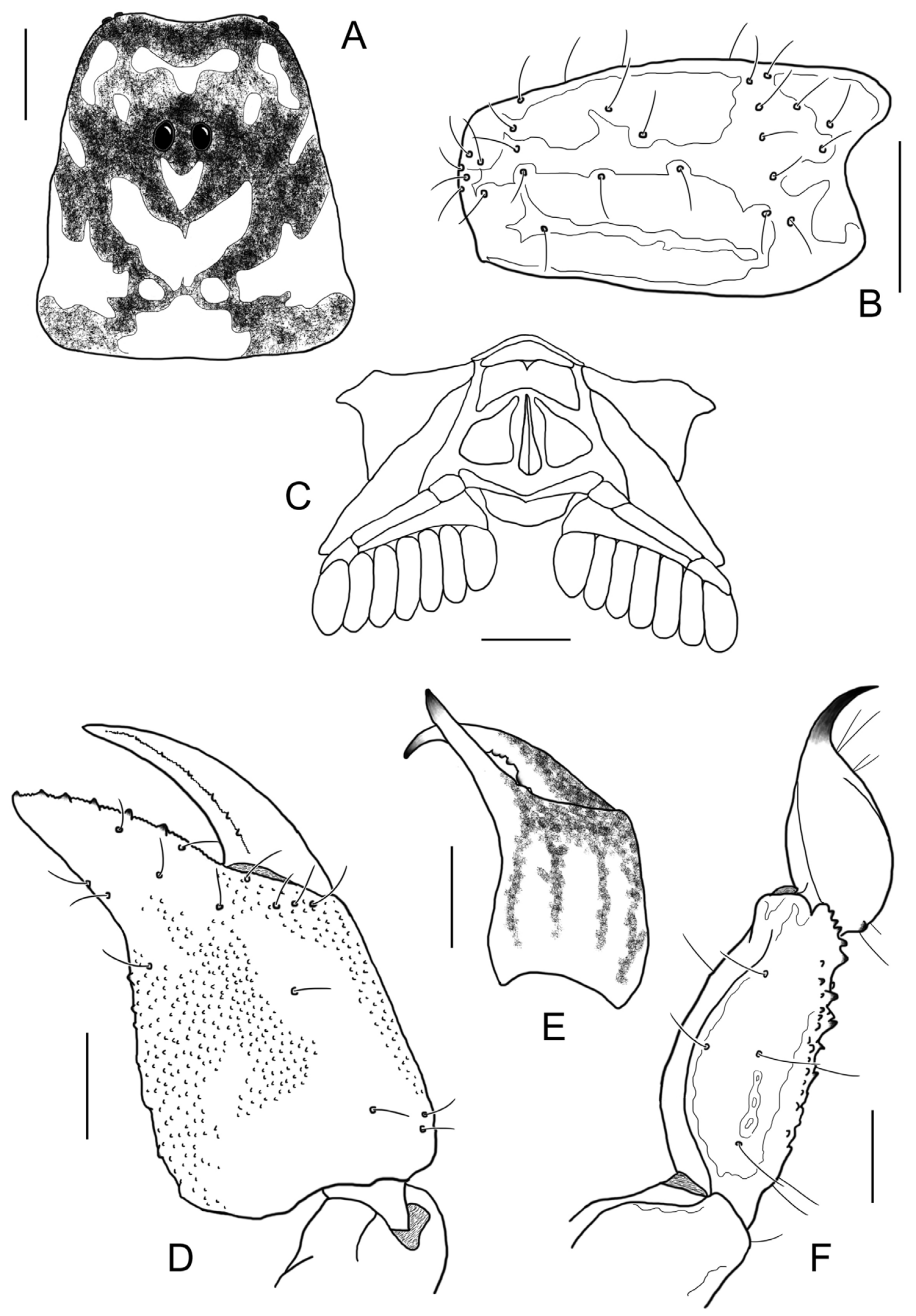
*Auyantepuia aurum* sp. n. male holotype. **A** Carapace **B** Patella, external aspect **C** Sternum, genital operculum and pectines **D** Chela, dorso-external aspect **E** Chelicera **F** Metasomal segment V and telson, lateral aspect. Scale bars: 1 mm except chelicera 0.5 mm (**E**).

###### Relationships.

*Auyantepuia aurum* sp. n. can be readily distinguished from other species of the genus *Auyantepuia* and, in particular, from the three species occurring in the central and southern part of French Guiana, by the following main features:

– *A. fravalae* Lourenço, 1983 (described from Saut Pararé on Arataye river (Approuage tributary), and also found in Saül): (i) tergites with confluent yellowish spots forming a yellowish longitudinal median stripe (no stripe in *A. fravalae*), (ii) ventral side of segments I and II yellowish, without spots (brownish spots on ventral side of segments I to V and ventral side of segments I to II well pigmented in *A. fravalae*).

– *A. sissomi* Lourenço, 1983 (described from upper Oyapok): (i) general coloration reddish brown (yellowish in *A. sissomi*), (ii) metasomal tegument with medium size granulation on ventral side of segments III to V (only segment V is granulated ventrally in *A. sissomi*).

– *A. kelleri* Lourenço, 1997 (described from Cacao): (i) tergites with confluent yellowish spots forming a yellowish longitudinal median stripe (no stripe in *A. kelleri*), (ii) ventral side of segments I and II yellowish, without spots (all segments uniformly dark reddish in *A. kelleri*), (iii) ocular tubercle darker, almost black (clear in *A. kelleri*).

##### Auyantepuia fravalae

Taxon classificationAnimaliaScorpionesChactidae

Lourenço, 1983

Fig. 19

###### References.

Lourenço 1983, Lourenço 1997b, Fet et al. 2000, Soleglad and Fet 2005, Prendini and Wheeler 2005, Lourenço and Qi 2007, Ythier 2015.

###### Material.

Downstream from Saut Pararé on Arataye river, Approuague tributary, one male (holotype), MNHN-RS8505, J.P. Gasc coll., IV/V/1979. Downstream from Saut Pararé on Arataye river, at the base of *Astrocaryum paramaca*, one female (allotype), MNHN-RS-8506, J.P. Gasc coll., I/1981. Saül, under dead wood, one female, deposited in the MHNG, P.K. Moritz coll., VIII/1987.

###### Diagnosis.

Total length 28.8 mm for male holotype and 28.6 mm for female allotype. General coloration reddish brown. Carapace dark reddish brown with blackish spots around the ocular tubercle and on lateral edges of the carapace; ocular tubercle dark, almost black. Tergites reddish with several confluent lighter zones. Venter greyish yellow, the sternite VII darker; pectines and genital operculum yellow ochre. All metasomal segments reddish, slightly darker than the mesosoma; several reticular blackish spots on the ventral and lateral sides of segments I to V. Vesicle reddish yellow with several darker spots corresponding to granules; basis of aculeus reddish and tip of aculeus reddish black. Chelicerae dark yellowish with blackish spots starting at the basis of fingers and spreading along the chelicerae; fingers reddish. Pedipalps reddish with several longitudinal blackish spots on the three segments (femur, patella, and chela). Legs light yellow with several diffuse light brown spots. Carapace with a fine granulation with bigger granules on the anterior part; anterior edge very slightly concave, almost straight. Tergites with a medium size granulation, especially on the posterior part. Pectinal tooth count 8–8 in both sexes. Sternites smooth with spiracles rounded in shape. Ventral side of metasomal segments IV and V with medium size granulation on IV, important and spinoid on V; dorsal carinae weakly marked on segments I to IV; latero-dorsal carinae well-marked on segments I to IV and weakly marked on V; other carinae absent. Vesicle large and flattened, with medium size granulation on ventral and lateral sides; aculeus short. Pedipalp femur with four almost complete carinae; patella and chela with vestigial carinae; dorsal and internal sides of femur granular; chela strongly granulated dorsally, only few scattered granules internally; dentate margins on movable fingers with six rows of granules separated by bigger granules.

**Figure 19.**
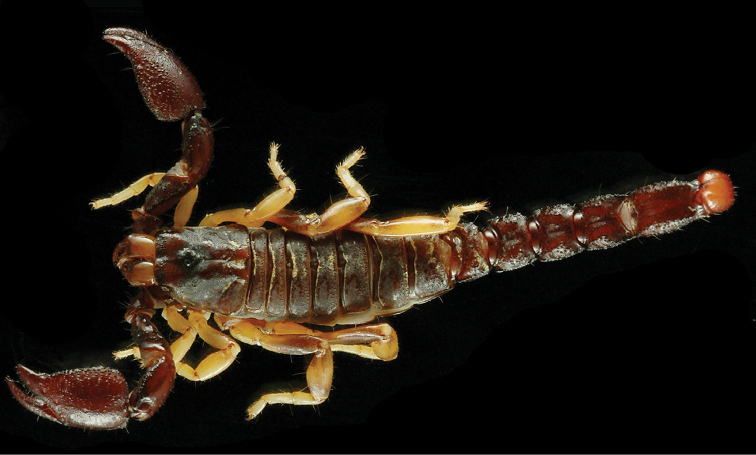
*Auyantepuia fravalae*, male holotype from Saut Pararé (photo MNHN / E.-A. Leguin).

##### Auyantepuia gaillardi

Taxon classificationAnimaliaScorpionesChactidae

Lourenço, 1983

###### References.

Lourenço 1983, Fet et al. 2000, Soleglad and Fet 2005, Prendini and Wheeler 2005, Lourenço and Qi 2007, Ythier 2015.

###### Material.

St Jean du Maroni, one male (holotype), one female (allotype) and six females (paratypes), MNHN-RS-3311, R. Benoist coll., XII/1913. St Jean du Maroni, four females (paratypes), MNHN-RS-3307, R. Benoist coll., III-IV/1914. St Jean du Maroni, one male (paratype), MNHN-RS-3326, R. Benoist coll., 18/XII/1913.

###### Diagnosis.

Total length 25.2 mm for male holotype and 26.9 mm for female allotype. General coloration reddish yellow. Carapace light reddish with yellowish spots on the posterior and lateral edges of the carapace; ocular tubercle blackish. Tergites yellowish with several confluent darker spots, greyish. Venter pale yellow, the sternite VII darker; pectines and genital operculum yellow ochre. All metasomal segments uniformly reddish, slightly darker than the prosoma. Vesicle of same coloration as metasomal segment V; aculeus dark reddish. Chelicerae uniformly yellowish; tip of fingers reddish. Pedipalps reddish, the femur slightly yellowish. Legs uniformly pale yellow. Carapace with a fine granulation in males, smooth in female; anterior edge very slightly concave, almost straight. Tergites with a fine granulation similar to the one on carapace in males, smooth in females. Number of pectine teeth ranging from seven to eight in male and six to seven in female. Sternites smooth with spiracles rounded in shape. Metasomal segment V with spinoid granulation ventrally; dorsal and latero-dorsal carinae on segments I to V weakly marked; other carinae absent. Vesicle with few scattered granules ventrally, other sides smooth; aculeus short. Pedipalp femur with dorsal internal, dorsal external and ventral internal carinae well-marked; internal side granular; patella and chela with vestigial carinae; chela weakly granular dorsally; dentate margins on movable fingers with six rows of granules separated by bigger granules.

##### Auyantepuia kelleri

Taxon classificationAnimaliaScorpionesChactidae

Lourenço, 1997

###### References.

Lourenço 1997b, Fet et al. 2000, Soleglad and Fet 2005, Prendini and Wheeler 2005, Lourenço and Qi 2007, Ythier 2015.

###### Material.

Cacao, one female (holotype), deposited in the MHNG, W. Lourenço leg., II/1989.

###### Diagnosis.

General coloration brownish. Carapace brownish with darker spots on the anterior part of the carapace; posterior part and furrows lighter; ocular tubercle light. Tergites brownish with confluent yellowish spots. Venter yellowish brown. All metasomal segments uniformly dark reddish. Vesicle reddish with basis of aculeus reddish and tip of aculeus reddish black. Chelicerae yellowish red with greyish spots; fingers yellowish with reddish teeth. Pedipalps dark reddish, chela reddish. Legs yellowish with slightly variegated greyish spots. Carapace without granules, almost smooth; anterior edge very slightly concave. Tergites with few minute and scattered granules. Pectinal tooth count 6–6 in female. Sternites smooth with spiracles rounded in shape. Metasomal segment V with spinoid granulation ventrally; dorsal and latero-dorsal carinae on segments I to V weakly marked; other carinae absent. Vesicle flattened with few scattered granules ventrally; aculeus of medium size. Pedipalp femur with dorsal internal, dorsal external and ventral internal carinae well-marked; internal side granular; patella and chela with vestigial carinae; chela weakly granulated dorsally and with few scattered granules internally; dentate margins on movable fingers with five rows of granules separated by bigger granules.

##### Auyantepuia laurae

Taxon classificationAnimaliaScorpionesChactidae

Ythier, 2015

Fig. 20

###### References.

Ythier 2015.

###### Material.

Near Saut Sabbat, 50 km South of Mana and 50 km East of Saint-Laurent-du-Maroni, under wood log, one female (holotype) and two females (paratypes), deposited in the MNHN, E. Ythier & G. Roy coll., I/2015. Mana, path of the Forêt des Sables Blancs, one female, deposited in the EYPC, EY0097, J. Chevalier & B. Tan coll., 08/VII/2017.

###### Diagnosis.

Size ranging from 27.5 to 28.2 mm in total length for the females. General coloration reddish brown. Carapace reddish yellow, intensely marked with brownish variegated spots around the ocular tubercle and on the anterior and posterior edges of the carapace; ocular tubercle darker, almost black. Tergites reddish brown with confluent reddish yellow spots, on the sides and the middle of tergites, without forming a longitudinal stripe. Venter and sternites yellowish to reddish yellow; sternum reddish yellow with darker spots; genital operculum reddish yellow; pectines pale yellow. Metasomal segments reddish yellow, marked with variegated brownish spots on lateral and dorsal sides of segments I to V and on ventral side of segments IV and V; ventral side of segments I to III yellowish, without spots. Vesicle reddish yellow with basis of aculeus blackish and tip of aculeus reddish. Chelicerae yellowish, with variegated dark brown spots; fingers reddish yellow with dark brown spots at their basis, reddish teeth. Pedipalps reddish brown, with longitudinal dark brown spots. Legs yellowish, intensely marked with brownish spots. Carapace lustrous and acarinate, with some minute punctations; furrows shallow; anterior edge emarginated. Tergites acarinate, almost smooth and shiny, with only minute granulations on their posterior edges. Pectinal tooth count 5–6 to 6–6 in females. Sternites smooth and shiny, VII acarinate; spiracles rounded in shape. Only metasomal segments IV and V longer than wide; metasomal tegument almost lustrous, without granulation, and with a few punctations; segment V with spinoid granulation ventrally, weakly marked; carinae on segments I to V vestigial or absent; only dorso-lateral carinae are weakly marked on segments I to IV. Pedipalp femur with dorsal internal, dorsal external and ventral internal carinae moderately marked; internal side weakly granular; other sides smooth; patella smooth, with vestigial carinae; chela weakly granulated, almost smooth, with dorso-internal carina weakly marked; dentate margins on fixed and movable fingers with six rows of granules.

**Figure 20.**
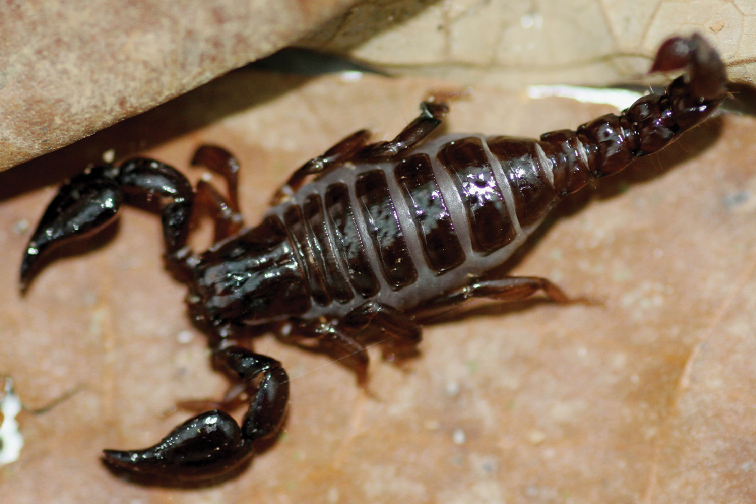
*Auyantepuia laurae*, female holotype from Saut Sabbat.

##### Auyantepuia sissomi

Taxon classificationAnimaliaScorpionesChactidae

Lourenço, 1983

Fig. 21

###### References.

Lourenço 1983, Fet et al. 2000, Soleglad and Fet 2005, Prendini and Wheeler 2005, Lourenço and Qi 2007, Ythier 2015.

###### Material.

Upper Oyapock, between Montaquoère junction and Dégrad Galoupa, equatorial forest, one female (holotype), MNHNRS3304 and one female (paratype), MNHNRS3309, mission E. Aubert de la Rüe coll., 10/I/1949.

###### Diagnosis.

Total length 26.2 mm for female holotype. General coloration yellowish. Carapace yellowish with light brown spots around the ocular tubercle and on the lateral edges of the carapace; posterior part and furrows lighter; ocular tubercle dark, almost black. Tergites greyish with several confluent lighter spots, yellowish, forming a longitudinal stripe. Venter yellow ochre. All metasomal segments reddish yellow, with greyish spots on lateral sides of I to V and on ventral side of III to V; ventral side of segments I and II without pigmentation. Vesicle reddish yellow; basis of aculeus reddish and tip of aculeus reddish black. Chelicerae yellowish with greyish spots; fingers yellowish with reddish teeth. Pedipalps reddish yellow with longitudinal light brown spots on the patella and chela, the dorsal side of the femur almost entirely covered with light brown spots; chela reddish. Legs pale yellow with several diffuse greyish spots. Carapace without granules, almost smooth; anterior edge very slightly concave. Tergites with few scattered fine granules. Number of pectine teeth ranging from six to seven in females. Sternites smooth with spiracles rounded in shape. Metasomal segment V with spinoid granulation ventrally; dorsal carinae weakly marked on segments I to IV; latero-dorsal carinae well-marked but incomplete on segments I to IV; other carinae absent. Vesicle flattened with few scattered granules ventrally; aculeus of medium size. Pedipalp femur with three carinae well-marked and almost complete; patella and chela with vestigial carinae; dorsal and internal sides of femur granular; dorsal side of chela granular, internal side with few scattered granules; dentate margins on movable fingers with six rows of granules separated by bigger granules.

**Figure 21.**
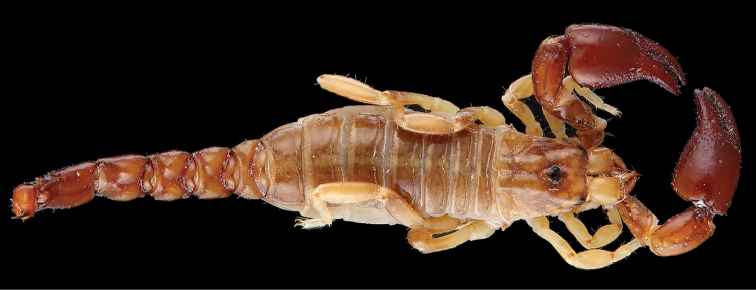
*Auyantepuia sissomi*, female holotype from upper Oyapock (photograph MNHN / E.-A. Leguin).

#### Genus *Broteochactas* Pocock, 1893

##### Broteochactas delicatus

Taxon classificationAnimaliaScorpionesChactidae

(Karsch, 1879)

Fig. 22

###### References.

Karsch 1879, Pocock 1897, Lourenço 1983, Fet et al. 2000, Lourenço 2002a, Soleglad and Fet 2005, Prendini and Wheeler 2005, Stockmann and Ythier 2010.

###### Material.

Camopi, Oyapock Valley, one female, MNHNRS3300, mission E. Aubert de la Rüe coll., 20/XII/1948. Camopi, Oyapock Valley, one male and two females, MNHNRS3392, mission E. Aubert de la Rüe coll., 18/XII/1948. St Georges de l’Oyapock, one female, W. Lourenço leg., 20/VIII/1982. Inini, Institut Pasteur Station, in forest, one female, MNHNRS6271, J.P. Gasc coll., 16/VII/1972. Inini, Institut Pasteur Station, one female, MNHNRS6273, J.P. Gasc coll., 13/VII/1972. Saül, six males and six females, MNHNRS5287, A.S. Balachowsky coll., 27/X/1969. Saül, inselberg Dashine, under barks on the ground from the standing dead tree, one male, deposited in the MNHN, J.P. Mauries coll., 05/IV/1997. Saül, Gros Arbres trail, two females and eleven immatures, deposited in the RNA, J. Chevalier, B. Tan & R. Legallic coll., 23/VIII/2017. Saül, Belvédère, one female and four immatures, deposited in the RNA, J. Chevalier, B. Tan & R. Legallic coll., 21–22/VIII/2017. Saut Sabbat, one female and 17 immatures, MNHNRS8202, D. Kopp coll., 9/VII/1976. Saut Sabbat, one female, MNHNRS8204, D. Kopp coll., 15/VII/1975. Trinité reserve, Aya River, UV collected, one male, deposited in the MNHN, C. Courtial coll., XII/2010. Nouragues reserve, inselberg, UV collected on a dead trunk, one immature male, deposited in the MNHN, C. Courtial coll., 06–09/XII/2013. Cacao, one male and one female, deposited in the MHNG, W. Lourenço leg., II/1989. Cacao, one female, deposited in the EYPC, EY0030, E. Ythier coll., 03–11/XI/2010. Cacao, beginning of Molokoi path, one male and one female, deposited in the EYPC, EY0104, E. Ythier coll., 03–10/XI/2006. Maripasoula, one female, deposited in the MHNG, Marty coll., X/1987. Mitaraka Massif, camp, one female, deposited in the MNHN, MNHN/PNI Guyane 2015, E. Poirier, P.H. Dalens & J. Touroult coll., 26/II/2015. Mana, path of the Forêt des Sables Blancs, two males and two females, deposited in the RNA, J. Chevalier & B. Tan coll., 08/VII/2017. Mana, path of the Forêt des Sables Blancs, three immatures, deposited in the RNA, J. Chevalier & B. Tan coll., 08/VII/2017. Guyane, one female, MNHNRS0755.

###### Diagnosis.

Species of moderate to large size when compared with the average size of the other species within the genus, ranging from 44 to 50 mm in total length. General coloration reddish to dark reddish. Carapace reddish with some light brown zones on the anterior edge and around the ocular tubercle; the areas between the anterior edge and the ocular tubercle as well as areas of furrows almost without pigmentation; ocular tubercle dark, almost black. Tergites reddish with several yellowish reticular diffuse spots on all tergites. Sternites yellowish with brown spots on lateral edges, especially on sternites VI and VII; pectines and genital operculum yellow ochre. Metasomal segments of same color as the tergites. Vesicle reddish yellow with basis of aculeus reddish and tip of aculeus reddish black. Chelicerae yellowish with longitudinal brown spots; basis of fingers and fingers dark brown. Pedipalps reddish with diffuse and reticular light brown spots on femur and patella. Legs yellowish with diffuse light brown spots. Carapace slightly emarginated, almost straight; almost smooth, with few minute granules only on lateral areas. Tergites almost smooth, with scattered minute granulations. Pectinal tooth count ranging from seven to nine in male and 7–7 in female. Sternites smooth; spiracles oval to round in shape. All metasomal segments with dorsal and latero-dorsal carinae well-marked; other carinae weakly marked or absent; segment V with spinoid granulations ventrally. Vesicle flattened, moderately granular; aculeus of moderate size compared to vesicle. Pedipalp femur with four well-marked carinae; patella with three carinae, chela with two carinae; femur and patella feebly granulated; chela well granulated on dorsal and internal sides, with granules arranged in four longitudinal wide series on external side; fingers about the same length as the chela and with dentate margins on movable fingers with six rows of granules, separated by stronger accessory granules.

**Figure 22.**
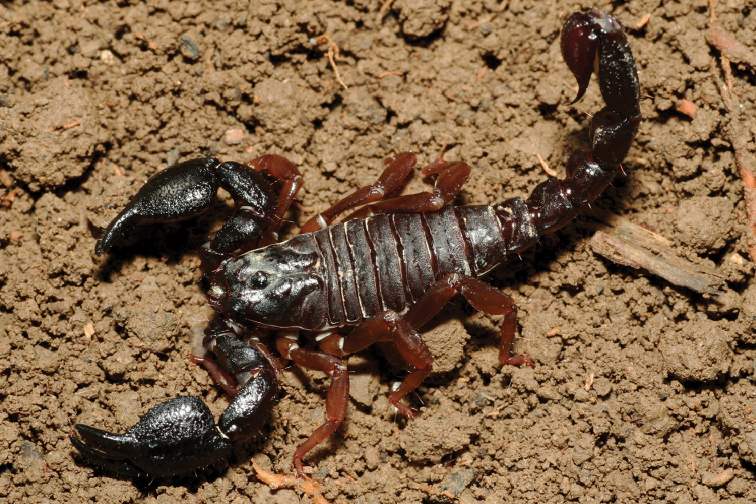
*Broteochactas delicatus*, male from Cacao.

#### Genus *Brotheas* C. L. Koch, 1837

##### Brotheas gervaisii

Taxon classificationAnimaliaScorpionesChactidae

Pocock, 1893

Fig. 23

###### References.

Pocock 1893a, Pocock 1897, Lourenço 1983, Lourenço 1997b, Fet et al. 2000, Lourenço 2002a, Stockmann and Ythier 2010.

###### Material.

Downstream from Saut Pararé on Arataye river, Approuague tributary, one female, MNHNRS7389, two males, MNHNRS7390, J.P. Gasc coll., IVV/1979. Cayenne, La Chaumière, one male, MNHNRS8507, J. Orvoen coll., V/1977. Cayenne, Mont Bourda, one female, deposited in the RNA, J. Chevalier & B. Tan coll., 09/VII/2017. Coswine river, under flooded wood log, two males and five females, MNHN-RS8297, J. Fretey coll., 16/V/1977. Upper Oyapock, Upper Camopi, Upstream Saül, Yanioué, one male, MNHNRS3295, mission E. Aubert de la Rüe coll., II/1949. Saül, one female, MNHNRS3320, mission E. Aubert de la Rüe coll. Saül, two females, MNHNRS5288, A.S. Balachowsky coll., 27/X/1969. Saül, one female, deposited in the MHNG, W. Lourenço leg., 23/VII/1987. Saül, one male juvenile, deposited in the MHNG, W. Lourenço leg., 16/VII/1986. Saül, Belvédère, one male, deposited in the RNA, J. Chevalier, B. Tan & R. Legallic coll., 21–22/VIII/2017. Saut Hermina, banks of Maroni River, three males, MNHNRS3316, H. Lourtau coll., 1901. St. Jean du Maroni, two males, one female and six immatures, MNHNRS3306, R. Benoist coll., 1914. Cacao, F-T-574, one male, deposited in the MHNG, T. Freitag coll., XII/1988. Cacao, one male and one female, deposited in the MHNG, W. Lourenço leg., II/1989. Cacao, one female, deposited in the MHNG, Chippaux coll., X/1983. Regina-St. Georges future road, DZ3, one male, deposited in the MHNG, Marty coll., I/1991. Trinité reserve, Aya river, UV collected, on the ground, three females, deposited in the MNHN, C. Courtial coll., XII/2010. Matoury, one female, deposited in the EYPC, EY0041, E. Ythier coll., 25/IX/2005. Matoury, one female and two immatures, deposited in the EYPC, EY0034, E. Ythier coll., 03–11/XI/2010. Fourgassier, one immature, deposited in the EYPC, EY0036, E. Ythier coll., 03–11/XI/2010. Rorota, one immature, deposited in the EYPC, EY0040, E. Ythier & G. Roy coll., 14–22/I/2015. Apatou, Crevette river, one male, deposited in the RNA, J. Chevalier & P. Gallier coll., 30/VI/2017. Awala Yalimapo, Kanawa path, three males, deposited in the RNA, J. Chevalier coll., XI/2017. Awala Yalimapo, Kanawa path, one male, deposited in the RNA, J. Chevalier coll., 03/VIII/2017. Mana, path of the Forêt des Sables Blancs, one male, deposited in the RNA, J. Chevalier & B. Tan coll., 08/VII/2017. Guyane, one male and one female, MNHNRS0741, Lafon coll., 1872. Guyane, one female, MNHNRS3308, Le Moult coll., 1910. Guyane, one male, MNHNRS0747. Guyane, one male and one female, MNHNRS0748. Guyane, one female, MNHNRS3011. Guyane, one female, MNHNRS0737, F. Geay coll., 1900. Guyane, one male, MNHNRS0739, Cavalier coll., 1889.

###### Diagnosis.

Species of small to moderate size when compared with the average size of the other species within the genus, ranging from 50 to 57 mm in total length. General coloration brown to reddish brown. Carapace reddish brown with several black spots corresponding to the granules; ocular tubercle dark, almost black. Tergites paler than the carapace, with distal edge blackish. Sternites reddish with a lighter area, yellowish, at the center of sternites IV and V; pectines and genital operculum yellow ochre. Metasomal segments of same color as the tergites. Vesicle reddish yellow with basis of aculeus reddish and tip of aculeus reddish black. Chelicerae yellowish with longitudinal brown spots, weakly marked; fingers reddish. Pedipalps reddish with blackish pigmentation on carinae. Legs yellowish with several brown spots. Carapace slightly emarginated, almost straight; almost smooth, with few minute granules only on lateral areas; punctate. Tergites almost smooth, with scattered minute granulations. Pectinal tooth count ranging from eight to eleven in male and six to nine in female. Sternites smooth, punctate; spiracles linear, elongated. All metasomal segments with dorsal, latero-dorsal and latero-ventral carinae well-marked; other carinae weakly marked or absent; intercarinal tegument punctate; segment V with spinoid granulations ventrally. Vesicle moderately granular; aculeus short compared to vesicle. Pedipalp femur with five well-marked carinae; patella with three carinae, chela with two carinae; femur and patella moderately granulated; chela well granulated on dorsal and internal sides; dentate margins on movable fingers with six slightly oblique rows of granules, separated by stronger accessory granules.

**Figure 23.**
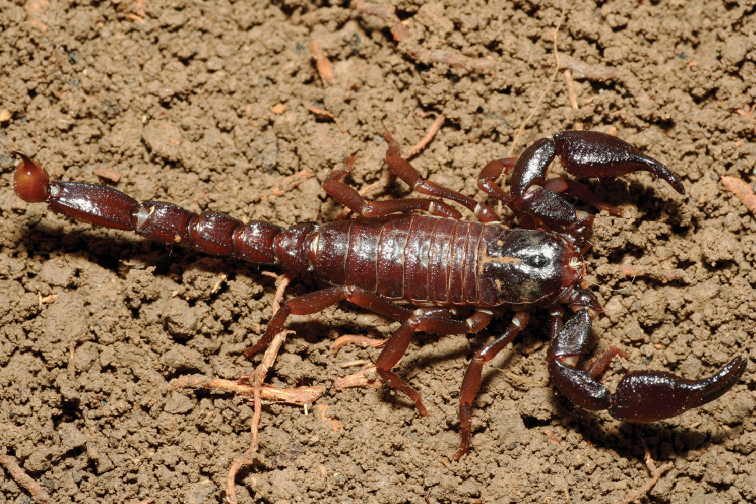
*Brotheas gervaisii*, male from Matoury.

##### Brotheas granulatus

Taxon classificationAnimaliaScorpionesChactidae

Simon, 1877

Fig. 24

###### References.

Simon 1877, Lourenço 1983, Lourenço 1997b, Fet et al. 2000, Lourenço 2002a, Stockmann and Ythier 2010.

###### Material.

Downstream from Saut Pararé on Arataye river, Approuague tributary, one male, MNHNRS6978, J.P. Gasc coll., VIIVIII/1970. Downstream from Saut Pararé on Arataye river, one male and two females, MNHNRS7390, J.P. Gasc coll., IVV/1979. Downstream from Saut Pararé on Arataye river, one male, MNHNRS8279, J.P. Gasc coll., 12/VII/1977. Downstream from Saut Pararé on Arataye river, at the base of *Astrocaryum paramaca*, six males and four females, MNHNRS-8508, J.P. Gasc coll., I/1981. Cayenne, one male and one female (types), MNHNRS-0761. Upper Approuague, Culebane river, in forest, one female, MNHNRS3302, De Floch coll., III/1946. Inini, Institut Pasteur Station, under a wood log, one male, MNHNRS6267, J.P. Gasc coll., 13/VII/1972. Inini, Institut Pasteur Station, one male, MNHNRS-6274, J.P. Gasc coll., 16/VII/1972. Inini, Institut Pasteur Station, one male, MNHNRS6275, J.P. Gasc coll., 14/VII/1972. Saül, one male, one female and six immatures, MNHNRS5288, A.S. Balachowsky coll., 27/X/1969. Saut Sabbat, one female, MNHNRS8203, D. Kopp coll., 9/VII/1976. Saut Sabbat, one male, MNHNRS8204, D. Kopp coll., 15/VII/1975. St. Elie (path), ORSTOM Station, one male and one female, MNHNRS7419, J. Lescure coll., 10/VII/1978. St. Jean du Maroni, five males and one female, MNHNRS3303, R. Benoist coll., 1916. Cacao, one female and one immature, deposited in the MHNG, W. Lourenço leg., II/1989. Cacao, one female, deposited in the MHNG, Chippaux coll., X/1983. Cacao, one male, deposited in the MHNG, T. Fretiag coll., XII/1988. Trinité reserve, inselberg, Aya River, five females and six immatures, deposited in the MNHN, C. Courtial coll., XII/2010. Trinité reserve, Aya River, one female and one male, deposited in the MNHN, C. Courtial coll., X/2009. Nouragues reserve, inselberg, UV collected, on a trunk, one female, deposited in the MNHN, C. Courtial coll., 06–09/XII/2013. Rorota, one female and two males, deposited in the EYPC, EY0026, E. Ythier coll., 28/IX/2005. Matoury, one female and 20 immatures, deposited in the EYPC, EY0035, E. Ythier coll., 03–10/XI/2006. Fourgassier, one male, deposited in the EYPC, EY0046, E. Ythier coll., 03–10/XI/2006. Tresor reserve, three males, deposited in the MNHN, C. Courtial coll., VI/2010. Cayenne and Maroni (?), two females, MNHNRS3296, M. Noirot coll. Cayenne and Maroni (?),one male and one female, MNHNRS-3312, E. Abonnenc coll. Cayenne and Maroni (?),one male, MNHNRS3318, M. Noirot coll. Guyane, one male, MNHNRS-0743, F. Geay coll., 1900. Guyane, four females, MNHNRS3313, M. Tartaire coll., 1921. Guyane, two females, MNHNRS0749.

###### Diagnosis.

Species of moderate to large size when compared with the average size of the other species within the genus, ranging from 58 to 68 mm in total length. General coloration brown to blackish brown. Carapace dark brown with several scattered black spots; ocular tubercle dark, almost black. Tergites of same color as the carapace; several paler confluent areas. Sternites brownish yellow, the sternite VII being darker; pectines and genital operculum dark yellowish. Metasomal segments of same color as the tergites; some blackish pigmentation over the carinae. Vesicle reddish yellow with basis of aculeus reddish and tip of aculeus reddish black. Chelicerae dark yellowish with reticular brown spots; basis of fingers and fingers dark browns. Pedipalps blackish brown; chela reddish with longitudinal blackish spots; fingers blackish with tip reddish. Legs yellowish brown with several diffuse dark spots. Carapace weakly emarginated, with strong granulation; ocular tubercle punctate. Tergites with strong granulation similar to the carapace, the tergites VI and VII with stronger granulation. Pectinal tooth count ranging from eight to eleven in male and seven to nine in female. Sternites punctate; spiracles linear, elongated. All metasomal segments with dorsal, latero-dorsal and latero-ventral carinae well-marked; ventral carinae absent on segment I and well-marked on other segments, with spinoid granules on segments IV and V; other carinae incomplete; intercarinal tegument moderately punctate. Vesicle with four vestigial carinae on ventral side; lateral and ventral sides weakly granulated; aculeus long compared to vesicle, wide at its basis. Pedipalp femur with five well-marked carinae; patella with four carinae; chela with vestigial carinae; dorsal and internal sides of femur well granulated; patella and chela moderately granulated except on internal side of chela where there are stronger granules; dentate margins on movable fingers with six slightly oblique rows of granules, separated by stronger accessory granules.

**Figure 24.**
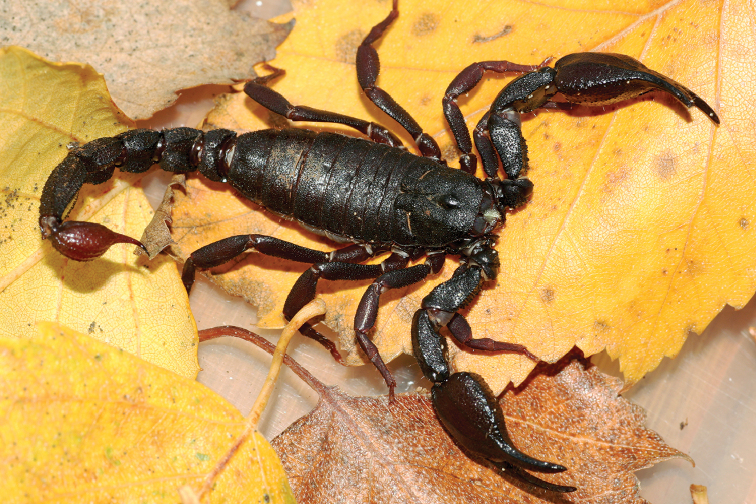
*Brotheas granulatus*, male from Fourgassier.

#### Genus *Guyanochactas* Lourenço, 1998

##### Guyanochactas flavus

Taxon classificationAnimaliaScorpionesChactidae

Lourenço & Ythier, 2011

Fig. 25

###### References.

Lourenço and Ythier 2011.

###### Material.

Roura-Cacao, Montagne Tortue, at the end of forested road of Bélizon, PK-27, under litter and/or log in trail, one male (holotype) and one female (paratype), deposited in the MNHN, J.P. Mauries & J.M. Betsch coll. 1/II/1992. Montsinéry-Tonnegrande, Anamites, one female (paratype), deposited in the MNHN, E. Ythier coll., XI/2010.

###### Diagnosis.

Total length 38.2 mm for male holotype and 35 mm for female paratypes. General coloration reddish yellow to pale yellow. Carapace yellowish with some reddish yellow zones. Tergites yellowish, slightly paler than carapace, with one longitudinal reddish yellow strip. Venter and sternites yellowish; pectines and genital operculum paler than sternites. Metasomal segments yellowish, with reddish yellow zones over carinae. Vesicle yellowish; aculeus reddish yellow at the base and reddish at the tip. Chelicerae yellowish, without spots; fingers reddish yellow with reddish teeth. Pedipalps yellow to reddish yellow with dark reddish zones over carinae. Legs yellow. Carapace slightly emarginated, with minute granulations and punctations; furrows shallow. Tergites acarinate, with only minute granulations and punctations. Pectinal tooth 9–9 in male and from 8–8 to 10–10 in female. Sternites smooth and punctate; VII acarinate; sternite III on female with a strong setation; spiracles oval in shape. Metasomal segments I to III wider than long; metasomal tegument with moderately marked granulations and a few punctations; segment V with spinoid granulations ventrally; carinae on segments I-V moderately to strongly marked; ventral carina vestigial on segment I, weakly marked on II, moderately marked on III and strongly marked on IV. Pedipalp femur with dorsal internal, dorsal external and ventral internal carinae moderately to strongly marked; ventral internal carina with spinoid granules; ventral external carina weakly marked; all aspects with minute granulations; patella with minute granulations and punctations; dorsal internal, ventral internal, ventral external and external carinae moderately marked; other carinae vestigial; chela with weakly to moderately marked granulations; ventral and dorsal median carina moderately marked; other carinae weakly marked; internal aspect with spinoid granules; dentate margins on movable and fixed fingers with five rows of granules.

**Figure 25.**
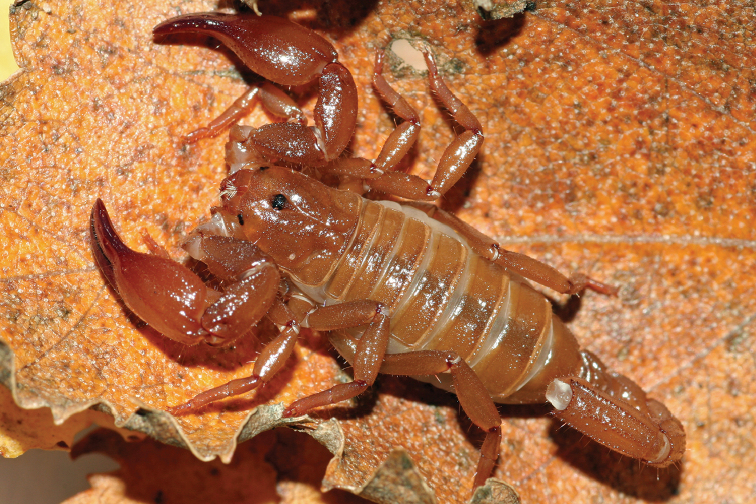
*Guyanochactas flavus*, female paratype from Anamites.

##### Guyanochactas gonzalezspongai

Taxon classificationAnimaliaScorpionesChactidae

(Lourenço, 1983)

Fig. 26

###### References.

Lourenço 1983, Fet et al. 2000, Lourenço & Pinto-da-Rocha 2000, Soleglad and Fet 2003, Prendini and Wheeler 2005, Stockmann and Ythier 2010, Lourenço and Ythier 2011.

###### Material.

AntecumePata, in forest, one male (holotype), MNHNRS6276, one male (paratype), MNHNRS6270, one male (paratype) MNHNRS6277, one male (paratype) MNHN-RS-6278 and one female (allotype), MNHNRS6266, J.P. Gasc coll., 18/VII/1972.

###### Diagnosis.

Species of large size compared to the average size of the other species within the genus (45.8 mm in total length for male holotype and 49.7 for female allotype). General coloration reddish yellow. Carapace dark reddish with several blackish spots, more concentrated in the central part of carapace; ocular tubercle dark, almost black. Tergites reddish, slightly paler than carapace, with several confluent lighter spots. Sternites reddish yellow, the last two ones darker; pectines and genital operculum yellow ochre. Metasomal segments reddish, with some blackish pigmentation on carinae. Vesicle reddish yellow; aculeus reddish at the base and reddish black at the tip. Chelicerae yellowish with longitudinal light brown spots; basis of finger and fingers dark brown. Pedipalps dark reddish with blackish pigmentation over carinae and chela fingers. Legs yellowish with diffuse light brown spots. Carapace slightly emarginated, the anterior third part strongly reduced; minute to medium granulation. Tergites with minute granulations; -several granules on posterior part, especially on sternite VII. Pectinal tooth 11–11 in male and 10–10 in female. Sternites smooth; spiracles oval in shape, almost rounded. All metasomal segments with dorsal, latero-dorsal and latero-ventral carinae well-marked; ventral carinae well-marked on segments III to V, weakly marked on II and absent on I; segment V with spinoid granulations ventrally. Vesicle flattened with ventral and lateral sides well granulated; aculeus short compared to vesicle. Pedipalp femur with five well-marked carinae; patella with four carinae; chela with weakly marked carinae; internal and dorsal sides of femur moderately granular; patella feebly granular; internal side of chela granular, other sides almost smooth; dentate margins on movable fingers with six rows of granules, separated by stronger granules.

**Figure 26.**
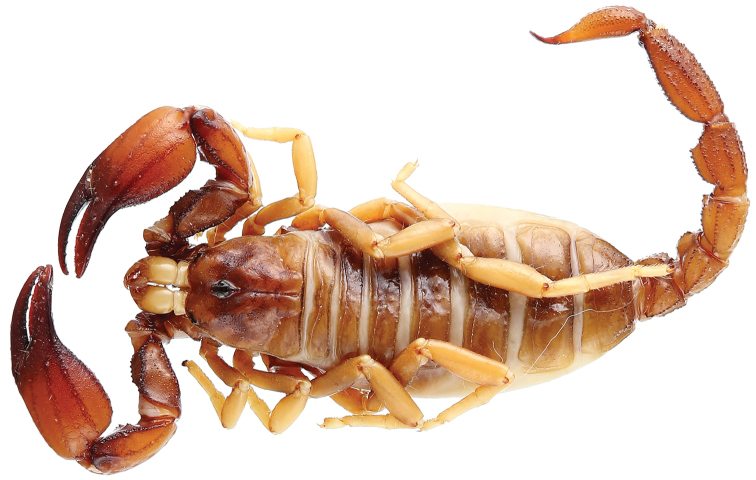
*Guyanochactas gonzalezspongai*, male holotype from Antecume-Pata (photograph MNHN / E.-A. Leguin).

#### Genus *Hadrurochactas* Pocock, 1893

##### Hadrurochactas cristinae sp. n.

Taxon classificationAnimaliaScorpionesChactidae

http://zoobank.org/D2AA5704-CB0C-462B-9487-688CA66B4327

Figs 27 , 28

###### Type material.

Roura, Stoupan, in litter, one male (holotype), deposited in the MNHN, Q. Uriot & S. Uriot coll., 2017.

###### Etymology.

The specific name honours Dr. Cristina Benros-Ythier, Romanèche-Thorins, France, in recognition of her support for the study of scorpions.

###### Diagnosis.

Total length 18.9 mm for male holotype (see morphometric values after the description). Coloration yellowish brown, densely spotted on the carapace, the tergites and the appendages. Mesosoma yellowish, densely spotted with confluent brown to dark brown spots, without yellowish longitudinal median stripe. Chelicerae yellowish with variegated brownish spots over the entire surface. Legs tarsal segments yellowish with diffused brownish pigmentation. Pectines with 9–9 teeth on male holotype; female unknown. Trichobothrial pattern of type C, neobothriotaxic (majorante) ‘major neobothriotaxy’.

###### Description based on male holotype.

**Coloration.** General coloration yellowish brown, densely spotted on the carapace, the tergites and the appendages. Prosoma yellowish with brownish variegated spots on anterior, lateral and posterior edges; eyes surrounded by black pigment. Mesosoma yellowish, densely spotted with confluent brownish spots, darker on the posterior edge of the tergites; the remaining yellowish coloration does not form a confluent longitudinal median stripe. Sternites yellowish, the VII slightly darker; coxapophysis and sternum yellowish; genital operculum and pectines pale yellow. Metasomal segments yellowish to reddish yellow with brownish spots. Telson reddish yellow with brownish spots; aculeus reddish. Chelicerae yellowish with variegated brownish spots over the entire surface; fingers yellowish almost entirely covered with brownish spots; teeth reddish yellow. Pedipalps reddish yellow with brownish longitudinal stripes on femur, patella, and chela; femur darker than the other segments. Legs with femur, patella, and tibia yellowish with brownish longitudinal stripes; basitarsus and telotarsus yellowish with diffused brownish pigmentation.

###### Morphology.

Carapace weakly granular to smooth; anterior margin very weakly emarginated; carinae absent; all furrows weakly pronounced; postero-median furrow finely granular; median ocular tubercle distinctly anterior to the center of the carapace; two pairs of small lateral eyes. All tergites with minute granulation and a few indistinct bigger granules on the posterior margin. Pectinal tooth count 9–9 in male holotype, fulcra absent. Sternites smooth and shiny except VI and VII which have some minute granulations; spiracles rounded; carinae absent; genital operculum longitudinally divided, each half with a sub-triangular shape. Dorsal carinae granular on metasomal segments I-IV, absent on segment V; dorsolateral carinae granular on all segments; ventrolateral and ventral carinae weakly pronounced or absent on all segments; dorsal surface smooth on all segments; lateral surfaces weakly granular to smooth on all segments; ventral surface smooth on segments I to IV; with some thin granules on V. Telson with small-sized spine-like granules and one larger spinoid granule under the aculeus; dorsal side smooth; aculeus relatively short and weakly curved. Pedipalp femur pentacarinate, moderately granular; patella and chela with weakly marked to unconspicuous carinae; fixed and movable fingers with seven rows of linear granules. Legs with long thin setae. Cheliceral dentition characteristic of the family Chactidae (Vachon, 1963). Trichobothriotaxy of type C, neobothriotaxic (majorante) ‘major neobothriotaxy’ (Vachon, 1974). Morphometric values (in mm) of the male holotype. Total length including telson, 18.9. Carapace: length, 2.3; anterior width, 1.3; posterior width, 2.4. Mesosoma length, 4.8. Metasomal segments. I: length, 0.9; width, 1.9; II: length, 1.2; width, 1.9; III: length, 1.2; width, 1.9; IV: length, 2.0; width, 1.9; V: length, 3.4; width, 2.2; depth, 1.4. Telson: length, 3.1; width, 0.9; depth, 0.6. Pedipalp: femur length, 1.9, width, 0.9; patella length, 2.3, width, 0.8; chela length, 3.7, width, 1.2, depth, 1.2; movable finger length, 2.2.

**Figure 27.**
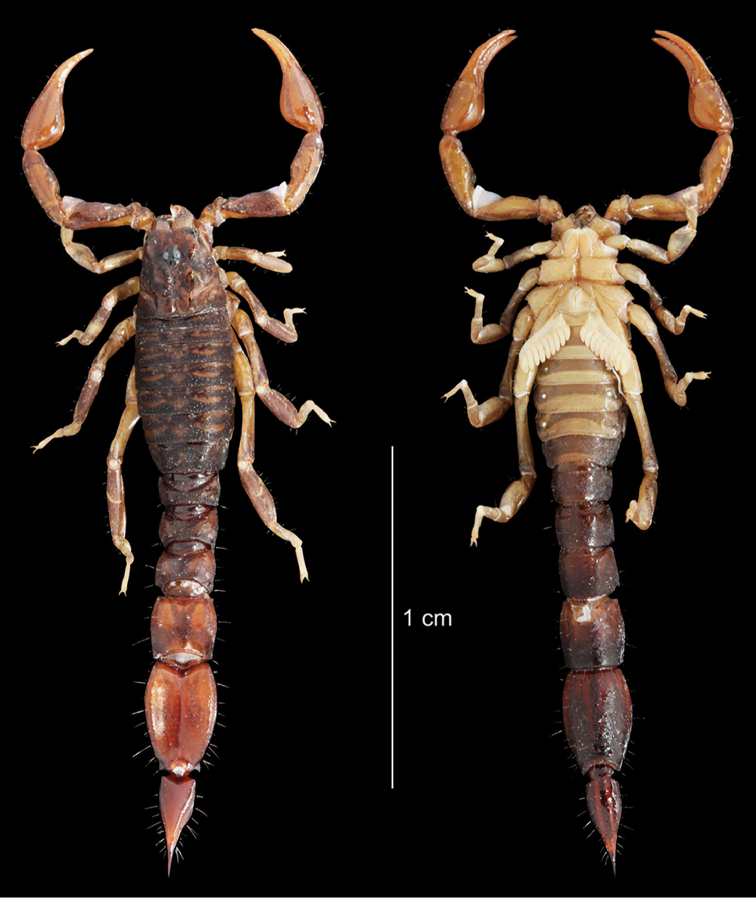
*Hadrurochactas cristinae* sp. n., male holotype from Roura. Habitus, dorsal and ventral aspect.

**Figure 28.**
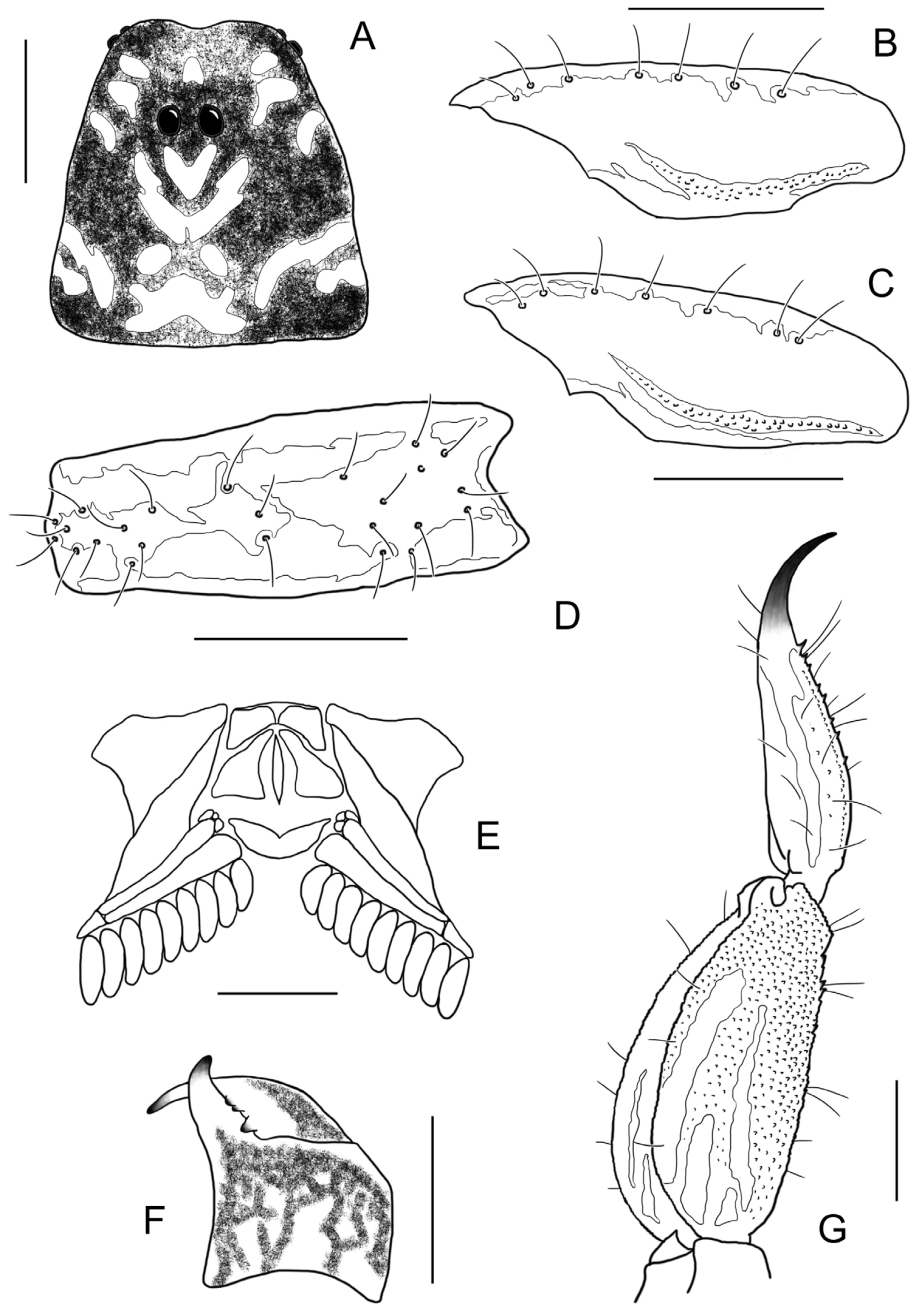
*Hadrurochactas cristinae* sp. n., male holotype. **A** Carapace **B–C** Patella, dorsal aspect of *H. cristinae* (**B**) and *H. schaumii* (**C**) **D** Patella, external aspect **E** Sternum, genital operculum and pectines **F** Chelicera **G** Metasomal segment V and telson, lateral aspect. Scale bars: 1 mm except chelicera 0.5 mm (**F**).

###### Relationships.

*Hadrurochactas cristinae* sp. n. can be readily distinguished from the other species of the genus *Harurochactas* and, in particular, from *H. schaumii* (the only other species described from French Guiana), by the following main features: (i) smaller general size (21.1 to 26.5 mm in total length for *H. schaumii*), (ii) male pectines with only 9–9 teeth (ranging from 10–11 *in H. schaumii*), (iii) general coloration yellowish brown (reddish brown in *H. schaumii*) without confluent yellowish longitudinal median stripe on mesosoma (reddish yellow longitudinal median stripe in *H. schaumii*), (iv) chelicerae yellowish with variegated brownish spots over the entire surface (without spots in *H. schaumii*), (v) legs tarsal segments yellowish with diffused brownish pigmentation (no pigmentation in *H. schaumii*), (vi) trichobothria on ventral side of pedipalp patella (V1 to V7) situated at different positions.

##### Hadrurochactas schaumii

Taxon classificationAnimaliaScorpionesChactidae

(Karsch, 1880)

Fig. 29

###### References.

Karsch 1880, Gonzaléz-Sponga 1978, Lourenço 1988b, Fet et al. 2000, Monod and Lourenço 2001, Lourenço 2002a, Stockmann and Ythier 2010, Lourenço 2010.

###### Material.

Downstream from Saut Pararé on Arataye River, Approuague tributary, one male and one female, MNHNRS7389, one female, MNHNRS7390, J.P. Gasc coll., IV/V/1979. Downstream from Saut Pararé on Arataye River, at base of *Astrocaryum paramaca*, two females, MNHNRS8509, J.P. Gasc coll., I/1981. Kaw, road to Kaw, Patawa, one female, deposited in the EYPC, EY0026, E. Ythier coll., 30/IX/2005. Cacao, beginning of Molokoi path, one male, deposited in the EYPC, EY0104, E. Ythier coll., 03–10/XI/2006. Mana, Laussat, white sand, leaf litter, pifall, two males, deposited in the MNHN, LA15-0284-12, LA15-0304-18, J. Orivel, M. Fichaux, Jackie & N. Milhomme coll., 01/X/2015. Mana, Laussat, white sand, leaf litter, pitfall, two immatures, deposited in the MNHN, LA15-0151-13, LA15-0223-18, J. Orivel, M. Fichaux, Jackie & N. Milhomme coll., 28/VIII/2015. Saint Eugène Research station, on the Courcibo stream, tributary of Sinnamary River, two males and two females, deposited in the MNHN, J.-C. de Massary leg.

###### Diagnosis.

Species of medium size when compared with the average size of the other species of the genus, ranging from 21.1 mm (male) to 26.5 mm (female) in total length. General coloration reddish brown. Carapace reddish with reddish brown spots. Tergites reddish brown with a confluent reddish yellow longitudinal median stripe. Venter and sternites light reddish brown; pectines yellowish. Metasomal segments reddish with brownish spots. Vesicle reddish; aculeus reddish at the base and black at the tip. Chelicerae yellowish without variegated spots over their entire surface, and with only a dark thin zone at the base of the fingers; fingers yellowish with reddish teeth. Pedipalps reddish brown with reticular brownish spots forming longitudinal stripes. Legs with femur, patella and tibia yellowish brown with dark brown spots; tarsal segments yellowish. Carapace densely covered with minute granulation with bigger granules in furrows and smooth areas mainly between the ocular tubercle and lateral eyes. Tergites with minute granulation in male, VII with bigger granules; tergites I-IV smooth and shiny in female. Pectinal tooth count ranging from 10–11 in male and from 8–10 in female. Sternites III/IV smooth and shiny in male, others with minute granulations; all sternites smooth and shiny in female; spiracles small and semi-oval in shape. Metasomal segments very strong in relation to the body; carinae only on dorsal and latero-dorsal sides; ventral side of metasomal segment I-IV in female and I-III in male smooth, the IV with fine granulation in male; segment V with ventral and lateral sides smooth in female and with granules in male. Telson smooth dorsally, granular with spinoid granules on lateral and ventral sides; aculeus weakly curved with a spinoid subaculear tooth. Pedipalp femur smooth in female and with granules on lateral sides in male, with five carinae; patella smooth and shiny, with three carinae; chela smooth and shiny, without granules and carinae; fingers about the same length as chela; fixed and movable fingers with seven rows of linear granules. Legs smooth, without granules and carinae.

**Figure 29.**
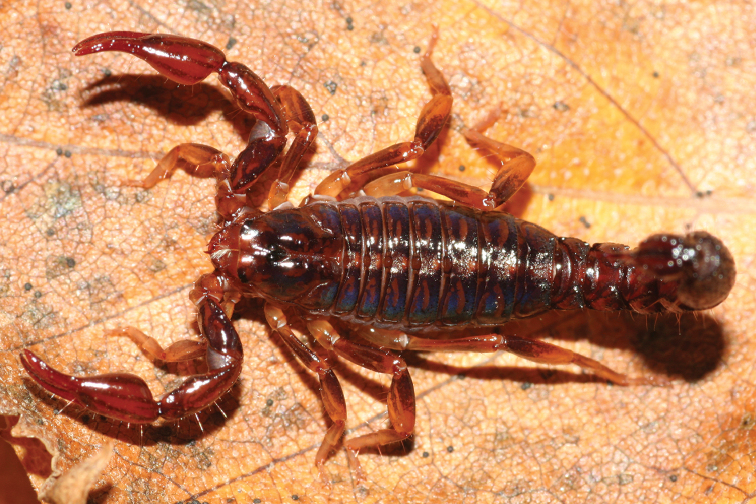
*Hadrurochactas schaumii*, male from Cacao.

#### Genus *Spinochactas* Lourenço, 2016

##### Spinochactas mitaraka

Taxon classificationAnimaliaScorpionesChactidae

Lourenço, 2016

Fig. 30

###### References.

Lourenço 2016a.

###### Material.

Mitaraka South, 640 m, one female (holotype), deposited in the MNHN, J.M. Betsch leg., 15/III/2001.

###### Diagnosis.

Total length 12.9 mm for female holotype. General coloration yellow to reddish yellow; only carapace and tergites are slightly marbled with brownish. Venter and sternites yellow; pectines pale yellow. Metasomal segments yellow. Chelicerae yellow without spots; fingers yellow with reddish teeth. Pedipalps yellow to reddish yellow. Legs pale yellow. Carapace lustrous and slightly punctate; carinae absent; furrows shallow. Tergites acarinate, with minute granulations only. Pectinal tooth count 6–6 in female. Sternites smooth and shiny; spiracles strongly reduced and rounded. Metasomal segments I to IV wider than long; metasomal tegument punctate except for some granulations on the ventral surface of segment V; ventral carinae absent from segments I to V; metasomal segments II to IV with dorsal and dorso-lateral carinae ending by a strong spinoid granule. Telson globular with a short aculeus; subaculear tooth or spine absent; some granulations present including on the dorsal side. Pedipalps slender with fingers strongly curved; femur with dorsal internal, dorsal external and ventral internal carinae moderately marked; ventral external carina vestigial; dorsal and ventral sides with minute granulations; internal side weakly granular; patella smooth and lustrous; dorsal internal, ventral internal, ventral external and external carinae weakly marked; other carinae vestigial; chela smooth and lustrous; carinae vestigial; internal side with granulations better marked on the base of fixed fingers; dentate margins on fixed and movable fingers with seven almost linear rows of granules, separated only by reduced internal accessory granules; edge of movable finger with three granules.

**Figure 30.**
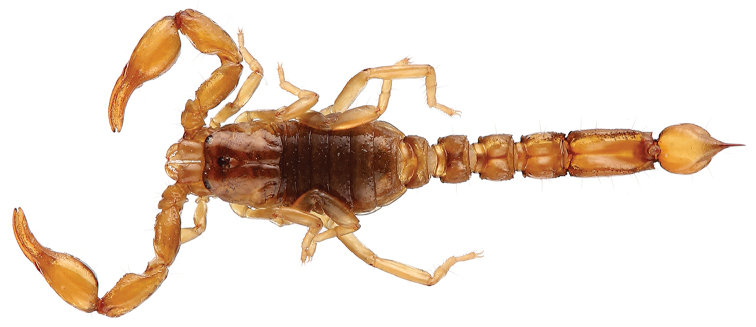
*Spinochactas mitaraka*, female holotype from Mitaraka massif (photograph MNHN / E.-A. Leguin; 2016 Elsevier Masson SAS).

### Family HORMURIDAE Laurie, 1896

#### Genus *Opisthacanthus* Peters, 1861

##### Opisthacanthus heurtaultae

Taxon classificationAnimaliaScorpionesHormuridae

Lourenço, 1980

###### References.

Lourenço 1980, Lourenço 1983, Fet et al. 2000, Lourenço and Fé 2003, Lourenço 2017.

###### Material.

Degrad Saramaca, surroundings of Kourou, forest patches of Degrad path, one male (holotype), MNHN-RS-8085, mission M. Boulard & P. Pompanon coll., 18/VIII/1975.

###### Diagnosis.

Total length 63.9 mm for male holotype. General coloration reddish brown. Carapace dark brown with some lighter zones; ocular tubercle slightly darker. Tergites dark brown with some lighter zones in the middle of tergites. Sternites greyish yellow, the VII darker; pectines and genital operculum greyish yellow, lighter than the sternites. Metasomal segments dark reddish brown with some lighter zones forming reddish yellow spots. Vesicle yellowish, darker around the articulation with metasomal segment V; aculeus yellowish at the base and reddish at the tip. Chelicerae dark yellow with reddish fingers. Pedipalps reddish brown with some darker zones; fingers darker. Legs reddish brown with some lighter spots, yellowish. Carapace strongly emarginated, with moderate granulation. Tergites with moderate granulation, better marked posteriorly. Pectinal tooth count 11–10 in male. Sternites smooth; spiracles linear. Metasomal segments rounded, all carinae weakly marked; segment V with spinoid granulations ventrally. Telson elongate, without any carinae; aculeus quite short compared to vesicle. Pedipalp femur with five carinae, four well-marked and one vestigial, intercarinal space smooth; patella and chela with several big granules forming incomplete carinae; chela long and flattened, dorsal and ventral sides smooth and shiny, lateral sides with strong granulations; fingers smooth, movable finger with a basal lobe. Numerous reddish yellow setae on the body, pedipalps, legs, and vesicle.

**Figure 31.**
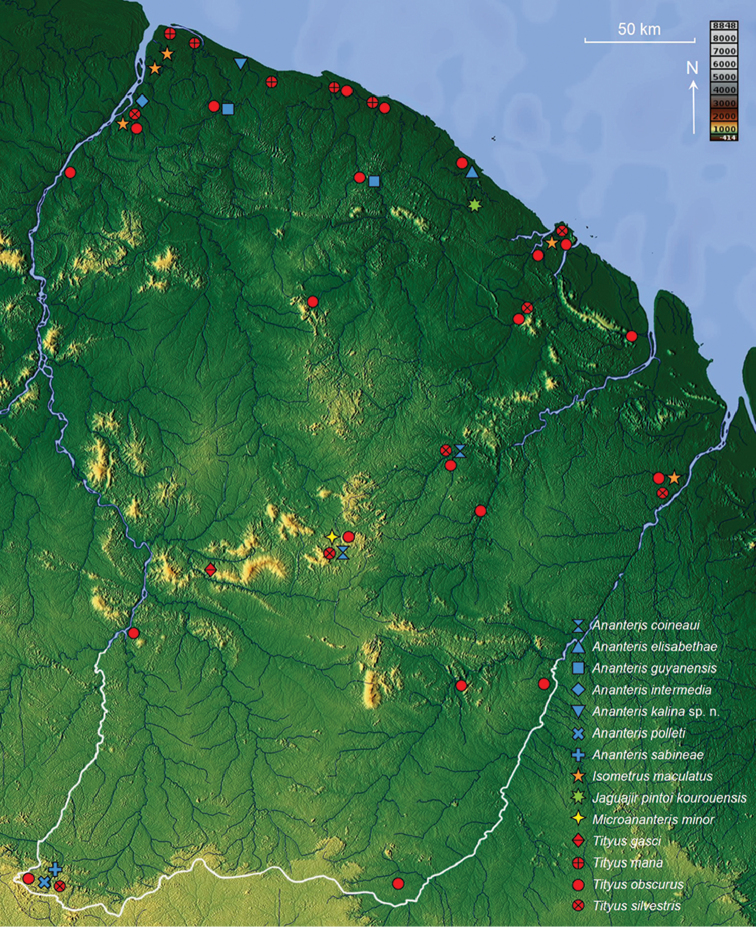
Topographic map of French Guiana showing the distribution of species of the family Buthidae.

**Figure 32.**
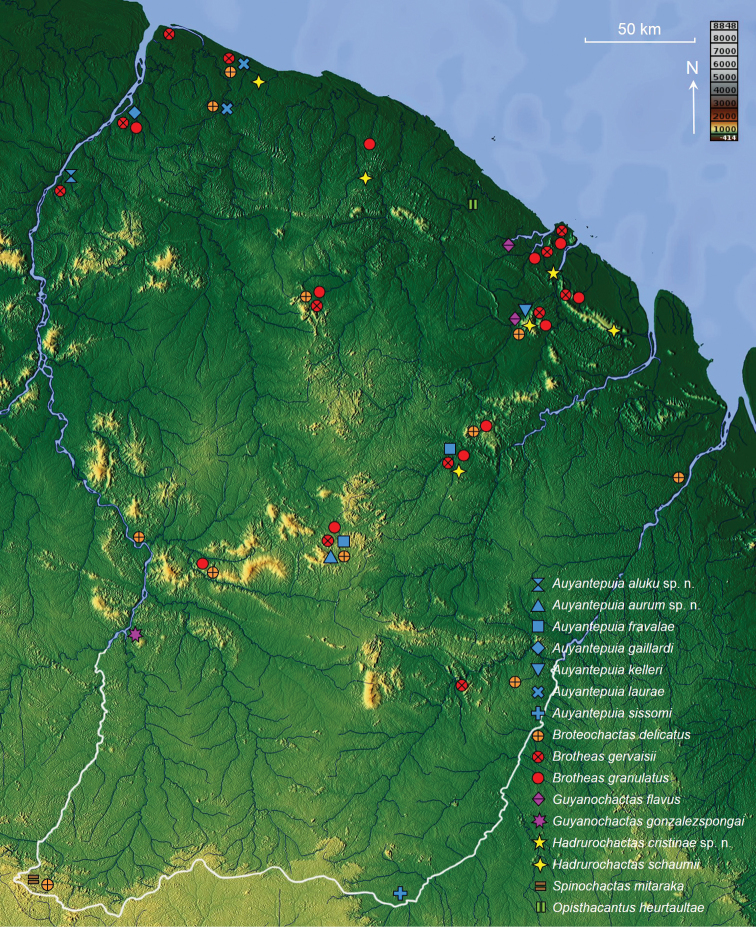
Topographic map of French Guiana showing the distribution of species of the families Chactidae and Hormuridae.

### Key to the species of scorpions described from French Guiana

The following key is proposed for the 30 species described from French Guiana and presented in this work. This key is based on previous keys proposed by Lourenço (1983, 2002b). This key must be considered as imperfect, provisional and susceptible to possible exceptions, hence it is to be used with caution and should not be the only tool for identifying a specimen. If there is any doubt, more thorough diagnoses presented in this paper or even detailed descriptions (see publications indicated in the references) should also be consulted.

**Table:** 

1	Sternum subtriangular; pedipalp tibia without ventral trichobothria	**Family Buthidae: 3**
–	Sternum subpentagonal	**2**
2	Retrolateral pedal spurs absent; anterior margin of carapace with a strong concavity; three lateral eyes; three ventral trichobothria on the tibia	**Family Hormuridae, Genus *Opisthacanthus* , *O. heurtaultae***
–	Retrolateral pedal spurs present; anterior margin of carapace without a strong concavity; two lateral eyes in most species; four to seven ventral trichobothria on the tibia	**Family Chactidae: 16**
3	Dentate margins of pedipalp chela fingers composed of 6/7 longitudinal rows of granules, without supernumerary granules	**4**
–	Dentate margins of pedipalp chela fingers composed of 8 to 17 oblique rows of granules	**12**
4	Presence of fulcra in the pectines	**Genus *Isometrus* , *I. maculatus***
–	Pectines without fulcra	**5**
5	Small species; very small pectines (pectinal tooth count 10–10) with distal teeth rounded	**Genus *Microananteris* , *M. minor***
–	Small to large species; pectines small to long (pectinal count ranging from 11 to 19), distal teeth not rounded	**Genus *Ananteris* : 6**
6	Chelicerae with reticular pattern	**7**
–	Chelicerae without reticular pattern	**10**
7	General coloration yellow to pale yellow; very small species (9 mm)	***A. intermedia***
–	Darker general coloration, brownish yellow; larger species (14–33mm)	**8**
8	Chelicerae reticular pattern incomplete, only a dark brown spot anteriorly at the base of fingers	***A. coineaui***
–	Reticular pattern complete, over the entire surface of the chelicerae	**9**
9	Small pectines with only 11 to 12 teeth in males	***A. polleti***
–	Pectinal tooth count 17–17 in males	***A. kalina* sp. n.**
10	General coloration pale yellow without spots or pigmented zones on the body and its appendages; movable fingers with six linear rows of granules	***A. elisabethae***
–	General coloration brownish yellow marbled with dark reddish brown spots; movable fingers with seven linear rows of granules	**11**
11	Movable fingers with one accessory granule present at the base of each row	***A. guyanensis***
–	Movable fingers with two accessory granules present at the base of each row	***A. sabineae***
12	Dentate margins of pedipalp chela fingers composed of 8/9 oblique rows of granules, with supernumerary granules present in the adults	**Genus *Jaguajir* , *Jaguajir pintoi kourouensis***
–	Dentate margins of pedipalp chela fingers composed of 12 to 17 oblique rows of granules, without supernumerary granules	**Genus *Tityus* : 13**
13	Small species ranging from 25 to 45 mm in total length with variegated pigmentation and a very rhomboidal subaculear tooth	**14**
–	Species of medium or large size, ranging from 63 to 100 mm in total length; pigmentation varying from yellowish to brown and black; spinoid subaculear tooth	**15**
14	Dorsolateral keels of metasomal segments I to IV with a moderate spinoid posterior granule	***T. mana***
–	Dorsolateral keels of metasomal segments I to IV without a spinoid posterior granule	***T. silvestris***
15	Species of medium size (63 mm in total length); coloration rather pale varying from yellowish to reddish brown or brownish, never black; basal middle lamellae of female pectines not dilated	***T. gasci***
–	Large species, ranging from 75 to 100 mm in total length; pigmentation blackish in the adult and yellowish/variegated in immature individuals; basal middle lamellae of female pectines dilated	***T. obscurus***
16	Spiracles linear; tarsus with two rows of spines	**Genus *Brotheas* : 28**
–	Spiracles oval or round; tarsus with setae or rows of spines	**17**
17	Tarsus with setae	**18**
–	Tarsus with rows of spines	**Genus *Guyanochactas* : 29**
18	Small scorpions, from 13 to 28 mm in total length; spiracles round	**19**
–	Scorpions of medium size, from 44 to 50 mm in total length; spiracles round to round/oval	**Genus *Broteochactas* , *B. delicatus***
19	Metasomal segments stocky in relation to the body, especially segments IV-V; fingers about the same length as chela hand	**Genus *Hadrurochactas* : 20**
–	Metasomal segments moderately stocky in relation to the body	**21**
20	General coloration reddish brown, with a confluent yellowish longitudinal median stripe over the tergites; chelicerae yellowish without spots; male pectinal tooth count ranging from 10–11	***H. schaumii***
–	General coloration yellowish brown, without confluent yellowish longitudinal median stripe; chelicerae with variegated brownish spots over the entire surface; male pectinal tooth count 9–9	***H. cristinae* sp. n.**
21	Pedipalp chela stocky; fingers shorter than chela hand	**Genus *Auyantepuia* : 22**
–	Pedipalp chela slender with fingers strongly curved; spinoid granules on dorsal and dorso-lateral carinae of metasomal segments II-IV	**Genus *Spinochactas* , *S. mitaraka***
22	Pedipalps with chelae weakly granulated, almost smooth	**23**
–	Pedipalps with chelae moderately to strongly granulated	**26**
23	Ventral side of several metasomal segments yellowish, without spots	**24**
–	Ventral side of all metasomal segments well pigmented, brownish to dark reddish	**25**
24	Posterior half of ventral side of segments I to IV yellowish, without spots; confluent yellowish longitudinal median stripe on tergites	***A. aluku* sp. n.**
–	Ventral side of metasomal segments I to III yellowish, without spots; no median stripe on tergites	***A. laurae***
25	Body, pedipalps, legs and chelicerae without variegated brownish spots	***A. gaillardi***
–	Body, pedipalps, legs and chelicerae marked with variegated brownish spots	***A. kelleri***
26	Ventral side of metasomal segments I to II yellowish, without spots; confluent yellowish longitudinal median stripe on tergites	**27**
–	Ventral side of all metasomal segments well pigmented, brownish to dark reddish; no median stripe on tergites	***A. fravalae***
27	General coloration reddish brown; metasomal tegument with medium size granulation on ventral side of segments III to V	***A. aurum* sp. n.**
–	General coloration yellowish; only metasomal segment V granulated ventrally	***A. sissomi***
28	Carapace and tergites strongly granular; general coloration blackish brown	***B. granulatus***
–	Carapace feebly granular; tergites punctate; general coloration reddish brown	***B. gervaisii***
29	General coloration reddish yellow with some blackish spots; chela fingers with six rows of granules	***G. gonzalezspongai***
–	General coloration pale yellowish, without any dark spots; chela fingers with five rows of granules	***G. flavus***

**Figure 33.**
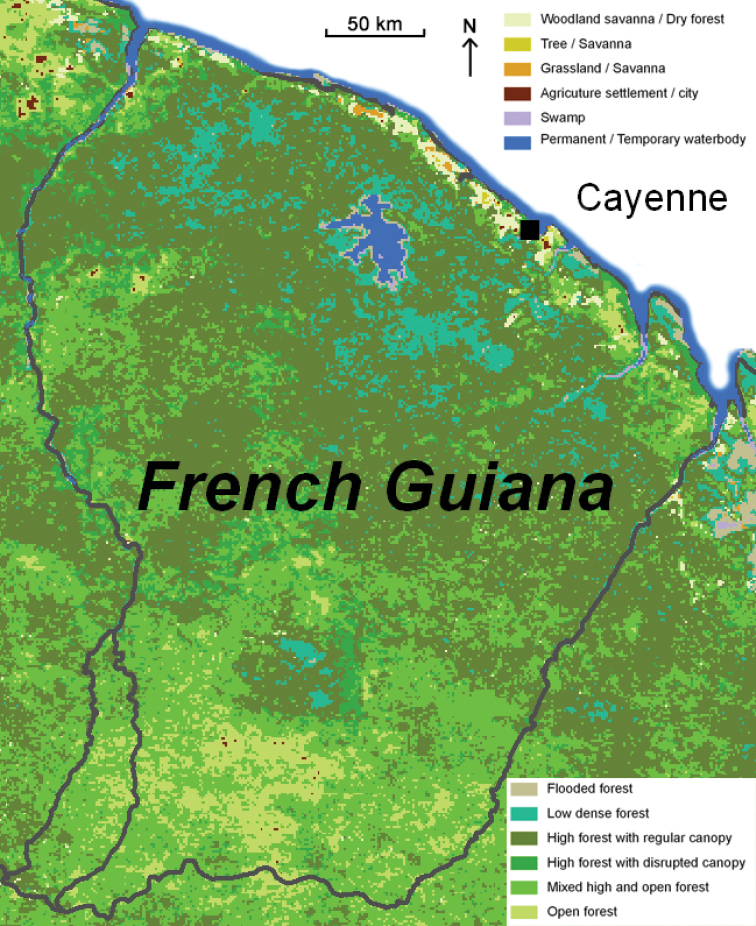
Vegetation map of French Guiana (modified from Gond et al. 2011).

**Figure 34.**
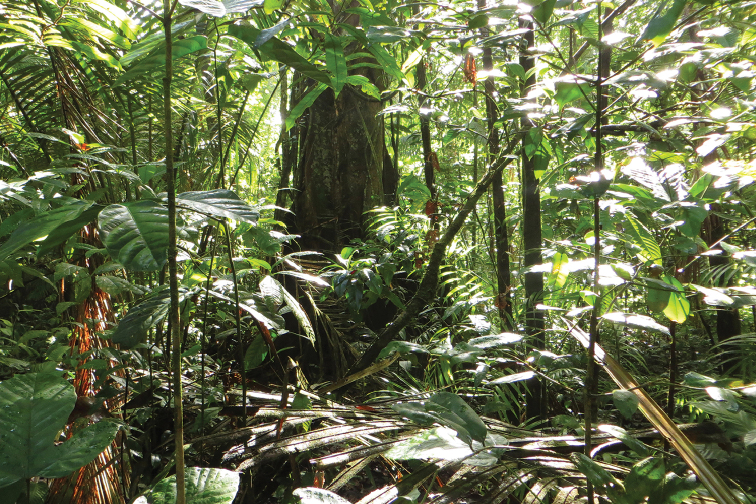
Lowland tropical rainforest habitat (Montsinéry-Tonnegrande, French Guiana).

**Figure 35.**
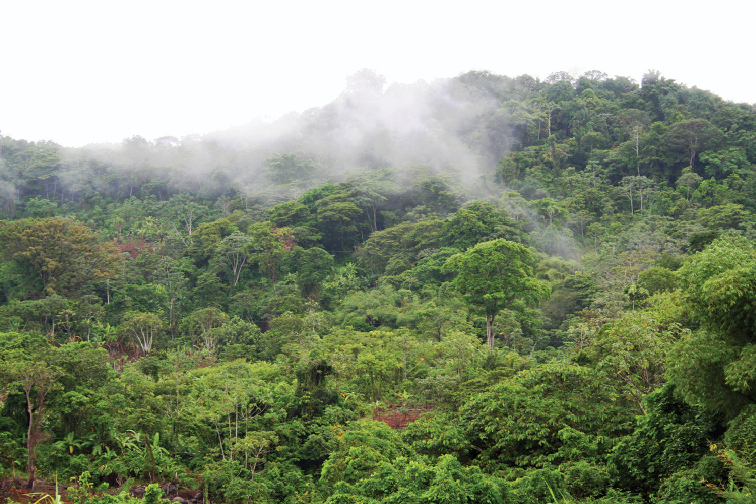
Altitude tropical rainforest habitat (Saül, French Guiana).

**Figure 36.**
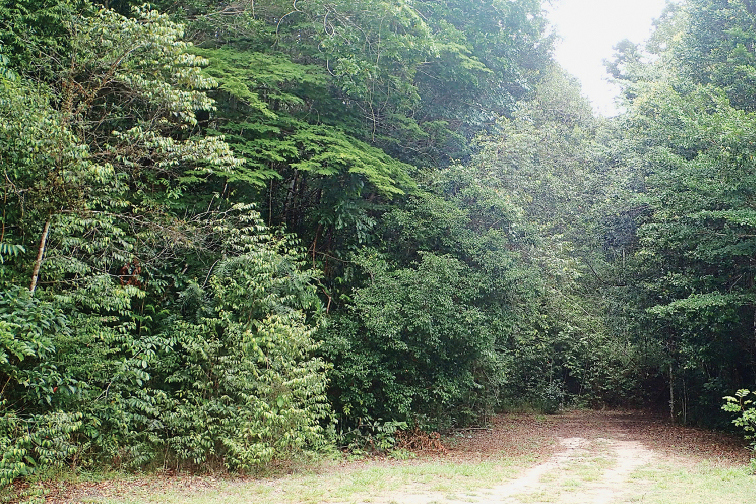
Coastal dry forest habitat (Mana, French Guiana). Photograph J. Chevalier.

**Figure 37.**
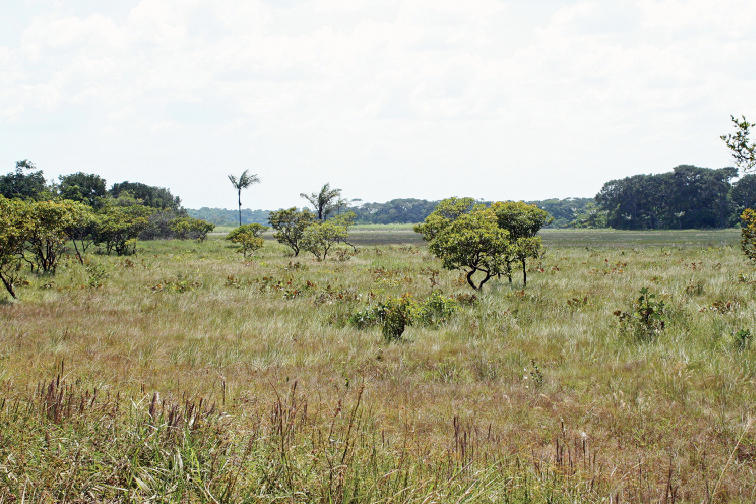
Coastal savanna habitat (Sinnamary, French Guiana).

## Supplementary Material

XML Treatment for Ananteris coineaui

XML Treatment for Ananteris elisabethae

XML Treatment for Ananteris guyanensis

XML Treatment for Ananteris intermedia

XML Treatment for Ananteris kalina

XML Treatment for Ananteris polleti

XML Treatment for Ananteris sabineae

XML Treatment for Isometrus maculatus

XML Treatment for Jaguajir pintoi kourouensis

XML Treatment for Microananteris minor

XML Treatment for Tityus (Tityus) gasci

XML Treatment for Tityus (Archaeotityus) mana

XML Treatment for Tityus (Atreus) obscurus

XML Treatment for Tityus (Archaeotityus) silvestris

XML Treatment for Auyantepuia aluku

XML Treatment for Auyantepuia aurum

XML Treatment for Auyantepuia fravalae

XML Treatment for Auyantepuia gaillardi

XML Treatment for Auyantepuia kelleri

XML Treatment for Auyantepuia laurae

XML Treatment for Auyantepuia sissomi

XML Treatment for Broteochactas delicatus

XML Treatment for Brotheas gervaisii

XML Treatment for Brotheas granulatus

XML Treatment for Guyanochactas flavus

XML Treatment for Guyanochactas gonzalezspongai

XML Treatment for Hadrurochactas cristinae

XML Treatment for Hadrurochactas schaumii

XML Treatment for Spinochactas mitaraka

XML Treatment for Opisthacanthus heurtaultae
